# A Cornucopia of Iridium Nitrogen Compounds Produced from Laser‐Ablated Iridium Atoms and Dinitrogen

**DOI:** 10.1002/chem.201905514

**Published:** 2020-04-30

**Authors:** Tony Stüker, Helmut Beckers, Sebastian Riedel

**Affiliations:** ^1^ Institut für Chemie und Biochemie Anorganische Chemie Freie Universität Berlin Fabeckstr. 34/36 14195 Berlin Germany

**Keywords:** density functional calculations, high oxidation states, matrix isolation, nitrogen fixation, transition metals

## Abstract

The reaction of laser‐ablated iridium atoms with dinitrogen molecules and nitrogen atoms yield several neutral and ionic iridium dinitrogen complexes such as Ir(N_2_), Ir(N_2_)^+^, Ir(N_2_)_2_, Ir(N_2_)_2_^−^, IrNNIr, as well as the nitrido complexes IrN, Ir(N)_2_ and IrIrN. These reaction products were deposited in solid neon, argon and nitrogen matrices and characterized by their infrared spectra. Assignments of vibrational bands are supported by ab initio and first principle calculations as well as ^14/15^N isotope substitution experiments. The structural and electronic properties of the new dinitrogen and nitrido iridium complexes are discussed. While the formation of the elusive dinitrido complex Ir(N)_2_ was observed in a subsequent reaction of IrN with N atoms within the cryogenic solid matrices, the threefold coordinated iridium trinitride Ir(N)_3_ could not be observed so far.

## Graphical abstract

**Iridium and nitrogen**: This work presents the last missing homoleptic dinitrogen and nitrido complexes of the platinum‐group metals and helps understanding the interaction of nitrogen with metals to facilitate nitrogen fixation. Dinitrogen activation, oxidation states and bond orders are discussed and give insight to the reactivity and electronic structure of these intriguing compounds.

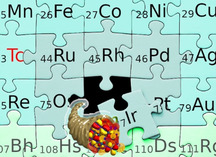

## Introduction

Molecular complexes combining nitrogen and platinum group metals (PGM), such as dinitrogen complexes L_*m*_M(N_2_)_*n*_ and polynitrido metal complexes L_*m*_M(N)_*n*_ have recently attracted much attention.^1^ Molecular dinitrogen complexes are of vivid interest in nitrogen fixation and reduction since 1966, when the first iridium dinitrogen complex was published, shortly after the first transition metal dinitrogen complexes [Ru(NH_3_)_5_N_2_]X_2_ with X=Br^−^, I^−^ and BF_4_^−^ were reported in 1965.^2^ The activation and weakening of the strong triple bond in the N_2_ molecule is facilitated by π‐back‐bonding from orthogonal d_*xz*_ and d_*yz*_ or even p orbitals into the antibonding π*‐orbitals of the N_2_ ligand.1a, 2c, 3 This effect is readily observable spectroscopically by a red‐shift of the N−N stretching mode compared to free dinitrogen in the IR spectra. All binary PGM dinitrogen complexes, except those of iridium, were investigated experimentally using matrix isolation techniques, where metal atoms are generated by thermal evaporation or laser ablation for Ru,^4^ Rh,^5^ Pd,^6^ Re,^7^ Os,^4^ and Pt.6b, 8 By these methods homoleptic dinitrogen complexes M(N_2_)_*n*_ can be prepared, which allow the investigation of metal–nitrogen bonding interactions independent of the influence of other ligands and thus give important insight into the bonding properties and mechanisms of dinitrogen activation.

Polynitrido metal complexes have recently attracted attention as the nitrido ligand facilitates high oxidation states. Examples are the group 6 complexes NM^+VI^F_3_ (with M=Cr, Mo and W)^9^ and the more recently predicted but so far unknown NIr^+IX^O_3_.1b The concept of “formal oxidation states” is a popular and important method of counting and assigning electrons to chemical elements in molecular and solid‐state structures.^10^ In recent years the range of compounds in high and unusual formal oxidation states has been expanded experimentally as well as theoretically. The so far highest experimentally attained formal oxidation state across all chemical elements is +IX of iridium in the [IrO_4_]^+^ cation.^11^ It was generated in the gas phase and detected using infrared photodissociation spectroscopy after it was predicted theoretically.^12^ But also compounds with iridium in the oxidation states of +VI and +VIII are scarce: Ir^+VI^F_6_, IrO_3_, Ir^+VI^(η^2^‐O_2_)(O)_2_ and Ir^+VIII^(O)_4_ are the only experimentally known examples.^13^ Nitrogen is the third most electronegative element and with a formal oxidation number of −3 it can increase the formal oxidation state of the metal center by three units, while occupying only a single coordination site. The problem associated with the N^3−^ ligand is that, compared to F^−^ and O^2−^, it is more easily oxidized by strong oxidizing metal centers, especially in complexes bearing metals in high oxidation states. Several binary transition metal nitrides were previously prepared by the reaction of laser‐ablated metal atoms with pure dinitrogen or dinitrogen diluted in rare gases, and subsequent deposition on a cold matrix support. Although this method mainly yields metal dinitrogen complexes, also molecular mono‐ and dinitrides of the platinum group metals, such as RuN and Ru(N)_2_,^4^ RhN and Rh(N)_2_,5b OsN and Os(N)_2_,^4^ and PtN8c were formed as well. So far, the only known binary molecular iridium nitrogen compound is the IrN molecule, first produced by laser ablation of iridium atoms in the presence of NH_3_ and characterized by optical/Stark spectroscopy.^14^ Subsequently, its spectral and bonding properties were studied further experimentally and theoretically.^15^ Furthermore, high‐pressure materials of the composition Ir_2_N, Ir(N)_2_ and Ir(N)_3_, respectively, are potentially (super) hard materials and their structural, electronic and mechanical properties were previously investigated theoretically^16^ and experimentally.^17^ These materials however contain quasi‐molecular N_2_^2−^ or N_2_^4−^ units rather than N^3−^.^18^

We have carried out reactions of laser‐ablated iridium atoms with dinitrogen molecules and studied the reaction products by matrix‐isolation IR spectroscopy. The photodecomposition of N_2_ molecules and the formation of N atoms induced by plasma radiation in the laser‐ablation process should also facilitate the formation of molecular binary iridium nitrides up to Ir^+IX^(N)_3_. These molecular binary iridium nitrides will allow to gauge the ability of the N atom to oxidize the iridium metal center and to investigate the nature of the chemical bonding independent of the influence of other ligands.

## Experimental and Computational Methods

### Matrix‐isolation experiments

^14^N_2_ (99.999 %, Linde) and ^15^N_2_ (98+ atom %, Campro) were premixed with neon or argon (both 99.999 %, Linde) in a stainless‐steel cylinder. The mixing vessel was connected to a stainless‐steel vacuum line connected to a self‐made matrix chamber by a stainless‐steel capillary. The gas mixture was then co‐deposited with laser‐ablated iridium atoms onto a CsI window (argon and dinitrogen matrices) or onto a gold plated copper mirror (neon matrices) and cooled to 4 K by using a closed‐cycle helium cryostat (Sumitomo Heavy Industries, RDK‐205D) inside the vacuum chamber. For the laser‐ablation, the 1064 nm fundamental of a Nd:YAG laser (Continuum, Minilite II, 10 Hz repetition rate, 35–50 mJ pulse^−1^) was focused onto a rotating iridium metal target through a hole in the cold window. Infrared spectra were recorded on a Bruker Vertex 70 spectrometer purged with dry air (argon and dinitrogen matrices) or a Bruker Vertex 80v with evacuated optical path (neon matrices) at 0.5 cm^−1^ resolution in the region 4000–430 cm^−1^ by using a liquid‐nitrogen‐cooled mercury cadmium telluride (MCT) detector. Far‐IR (FIR) spectra were recorded at a resolution of 0.5 cm^−1^ at the Bruker Vertex 80v equipped with a FIR multilayer mylar beam‐splitter (680–30 cm^−1^), a CsI window (>180 cm^−1^), and a liquid helium cooled bolometer. The matrix samples were irradiated by a mercury arc streetlamp (Osram HQL 250) with the outer globe removed. Wavelength selective irradiations in the visible spectrum were realized with OSRAM LEDs with typical powers between 5 and 10 watts.

### Electronic‐structure calculations

Density functional theory (DFT) calculations were performed using the TURBOMOLE 7.0.1 program package^19^ employing the GGA exchange‐correlation density functional BP86^20^ with the polarized quadruple‐ξ basis set def2‐QZVP^21^ which applies the Stuttgart‐Dresden effective core potential for iridium.^22^ All Coupled Cluster Single Double and perturbative Triple excitations (CCSD(T)) combined with Dunning's augmented correlation consistent polarized triple‐ξ basis sets aug‐cc‐pVTZ for nitrogen,^23^ and aug‐cc‐pVTZ‐PP combined with the ECP60MDF effective core potential for iridium^24^ were performed using the CFOUR 2.00beta software.^25^ State‐averaged complete active space self‐consistent field (SA‐CASSCF) calculations combined with Dunning's correlation consistent polarized valence triple‐ξ basis sets cc‐pVTZ^26^ and cc‐pVTZ‐PP^24^ for nitrogen and iridium and the effective core potential (ECP60MDF) for iridium were carried out for iridium dinitride using the Molpro 2019 software.^27^ The active space was chosen to consist of the molecular orbitals formed by the 2p(N), 5d(Ir) and 6s(Ir) atomic orbitals, yielding 15 electrons in 12 molecular orbitals. One calculation for each spin multiplicity, doublet, quartet and sextet was carried out employing the state‐averaging formalism in *C*_2*v*_ point group symmetry, including two states of each state symmetry (A_1_, B_1_, B_2_ and A_2_), resulting in eight states with equal weights of 0.125. Harmonic vibrational frequency calculations were carried out for all optimized structures analytically (BP86) or numerically (CCSD(T)). The decomposition pathways of Ir(N)_2_ and Ir(N)_3_ were analyzed by optimizing the geometries of the nitrides, the complexes formed by the rearrangement, and the transition states connecting both minima using the BP86 exchange‐correlation density functional with the application of the zeroth‐order regular relativistic approximation (ZORA)^28^ combined with the adapted version of the def2 basis set ZORA‐def2‐TZVPP for nitrogen and the segmented all‐electron relativistic contracted SARC‐ZORA‐TZVPP for iridium^29^ as implemented in ORCA 4.1.2.^30^ Additionally, the *meta*‐GGA M06‐L exchange correlation density functional^31^ was used for calculating the energy barriers associated with the decompositions of Ir(N)_2_ and Ir(N)_3_. The NBO and AIM analyses were carried out using wavefunctions obtained at the BP86/def2‐QZVP level of theory using NBO 7.0^31^ and Multiwfn 3.5,^33^ respectively. Because of the multitudes of combinations and the rapidly increasing computational challenges, compounds of the formula Ir_*x*_N_*y*_, with *y* and *x* greater than two are not explicitly considered.

## Results and Discussion

**Figure 1:**
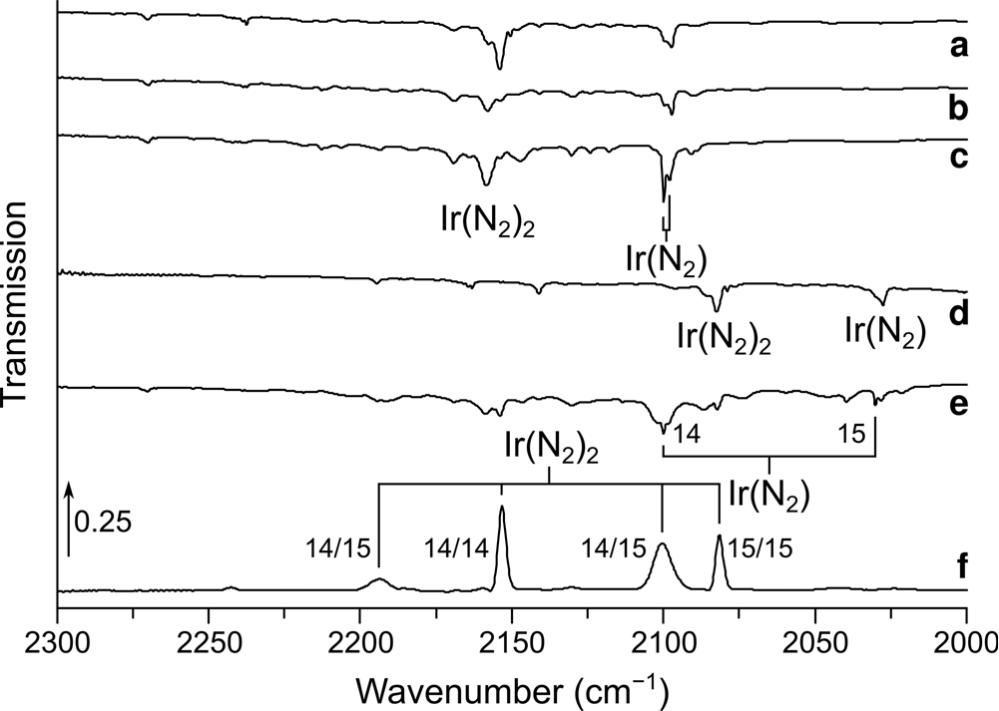
Infrared spectra in the 2000–2350 cm^−1^ region of the reaction products of laser‐ablated iridium atoms with 0.5 % ^14^N_2_ in neon after deposition (a), 10 min broadband irradiation (b), annealing to *T=*10 K (c), with 0.5 % ^15^N_2_ in neon after deposition (d), with 0.5 % of a 1:1 mixture of ^14^N_2_ and ^15^N_2_ in neon after 10 min of broadband irradiation and subsequent annealing of *T=*10 K (e) and with 10 % of a 1:1 mixture of ^14^N_2_ and ^15^N_2_ in neon after 10 min of irradiation with LED light *λ*=455 nm (f). Bands due to iridium nitrogen compounds and some selected ^14/15^N isotope patterns are indicated.

**Figure 2:**
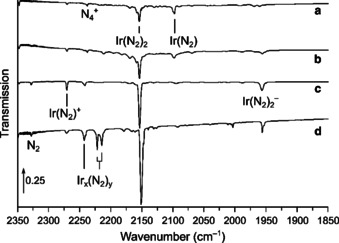
Infrared spectra in the 1850–2350 cm^−1^ region of the reaction products of laser‐ablated iridium atoms with 0.5 % (a), 3 % (b), 10 % (c) ^14^N_2_ in neon, and neat ^14^N_2_ (d). Bands due to iridium nitrogen compounds are indicated.

**Figure 3:**
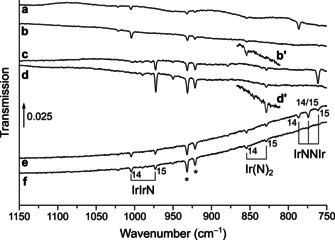
Infrared spectra in the 1150–750 cm^−1^ region of the reaction products of laser‐ablated iridium atoms in neon doped with 10 % ^14^N_2_ after deposition (a), after 10 min of 455 nm irradiation (b), with 10 % ^15^N_2_ in neon after deposition (c) after irradiation with 455 nm (d), as well as with 10 % of a 1:1 mixture of ^14^N_2_ and ^15^N_2_ in neon after deposition (e), and 10 min of irradiation with 455 nm (f). Bands due to iridium nitrogen compounds and some selected ^14/15^N isotope patterns are indicated. The sections showing the Ir(N)_2_ absorption in the spectra b and d are enhanced by a factor of 5 and tagged with b′ and d′.

**Figure 4:**
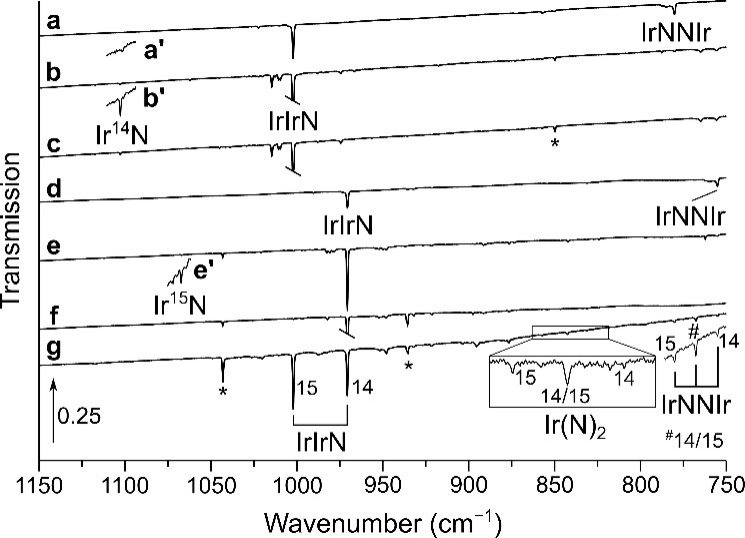
Infrared spectra in the 1150–750 cm^−1^ region of the reaction products of laser‐ablated iridium atoms with ^14^N_2_ after deposition (a), annealing to 35 K (b) and broadband irradiation (c), as well as with ^15^N_2_ after deposition (d), annealing to 35 K (e), broadband irradiation (f) and finally after deposition with a 1:1 mixture of ^14^N_2_ and ^15^N_2_ (g). Bands due to iridium nitrogen compounds and some selected ^14/15^N isotope patterns are indicated. Bands marked by an asterisk exhibit no isotopic shift and remained unassigned. The transmission of the bands shown in the sections a′, b′ and e′ are enhanced by a factor of 10, 10, and 15, respectively.

**Figure 5:**
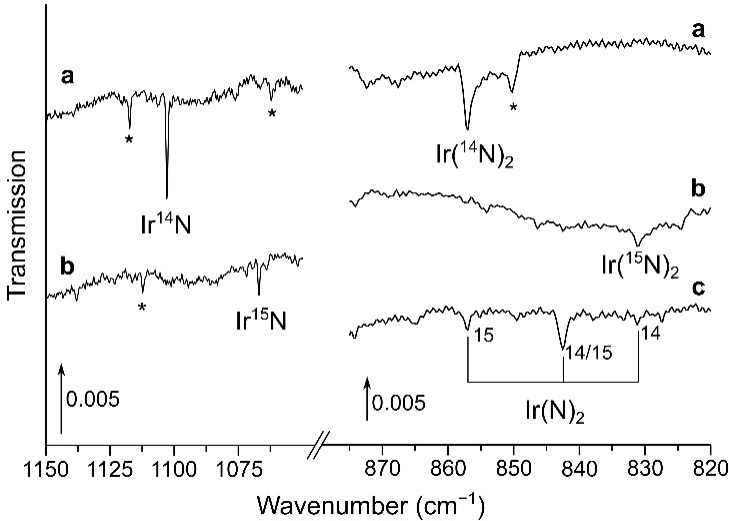
Infrared spectra in the 1150–1050 cm^−1^ and 875–820 cm^−1^ regions of the reaction products of laser‐ablated iridium atoms with ^14^N_2_ (a), ^15^N_2_ (b) as well as a 1:1 mixture of ^14^N_2_ and ^15^N_2_ (c). Bands due to iridium nitrogen compounds and some selected ^14/15^N isotope patterns are indicated. Bands marked with an asterisk exhibit no isotopic shift and remained unassigned.

Laser‐ablated iridium atoms were reacted with diluted dinitrogen in a vacuum chamber and the reaction products were subsequently deposited on a matrix support under cryogenic conditions and studied using IR spectroscopy. The experimental details are presented in the experimental section. The obtained products can be separated in two different sets: dinitrogen and nitrido complexes. The N−N stretching vibrations of the dinitrogen complexes occur in the region from 2350–1850 cm^−1^, and the Ir−N stretching vibrations of dinitrogen and nitrido complexes in the region below 1150 cm^−1^ (Table 1). The main absorptions that appeared in the N−N stretching region of the IR spectra are located at 2270.3, 2241.6, 2154.0, 2097.4, 1956.4 cm^−1^. They are assigned and labeled in Figures 1 and 2 to the dinitrogen complexes [Ir(N_2_)]^+^, NIr(N_2_), Ir(N_2_)_2_, Ir(N_2_) and [Ir(N_2_)_2_]^−^, respectively. In the Ir−N stretching region of these spectra absorptions of IrN, IrIrN, Ir(N)_2_ and IrNNIr were detected. These are indicated in Figures 3, 4 and 5 to bands at 1111.1, 1004.4, 853.5 and 786.5 cm^−1^, respectively. Additional bands were observed when the reaction products are deposited in neat nitrogen matrices (Table 2 and Figure 2, trace d). In the ^14^N_2_ matrices a band appeared at 2221.7 cm^−1^, accompanied by a matrix site at 2214.3 cm^−1^ (Figure 2), which is probably associated with clusters of Ir_*x*_(N_2_)_*y*_.

**Table 1:** Infrared absorptions (cm^−1^) and ^14^N_2_/^15^N_2_ isotopic ratios obtained from the reaction of laser‐ablated iridium atoms co‐deposited with dinitrogen diluted in neon at 4–5 K.

^14^N_2_	^15^N_2_	^14^N_2_ and ^15^N_2_	^14^N_2_/^15^N_2_ ratio	Assignment
2327.6	2250.1	2327.6, 2250.1	1.0344	N_2_ (perturbed)
2270.3	2194.8	2270.7, 2194.8	1.0344	Ir(N_2_)^+^
2241.6	2167.0	2241.6, 2167.0	1.0344	Ir_x_(N_2_)_*y*_
2237.4	2163.1	[a]	1.0343	N_4_^+^
2158.0	2086.1	[a], 2158.0 [a], 2086.1	1.0345	Ir(N_2_)_2_ (site)
2154.0	2082.4	2194.6, 2154.0 2100.3, 2082.4	1.0344	Ir(N_2_)_2_
2099.8	2030.0	2099.9, 2030.0	1.0344	Ir(N_2_)
2097.4	2027.9	[a]	1.0343	Ir(N_2_) (site)
1956.4	1890.3	1988.1, 1956.4 1912.9, 1891.5	1.0350	Ir(N_2_)_2_^−^
1111.1	1076.4	1111.1, 1076.4	1.0322	IrN
1004.4	972.8	1004.4, 972.8	1.0322	IrIrN
931.5	931.5	931.5		OIrO
921.6	921.6	921.6		OIrO (site)
853.5	827.7	853.3, 827.7	1.0312	Ir(N)_2_
786.5	761.6	786.5, 774.1, 761.6	1.0327	IrNNIr
		402.8, 397.4 393.1	1.0247	Ir(N_2_)_2_

[a] Not observed.

**Table 2:** Infrared absorptions (cm^−1^) and ^14^N_2_/^15^N_2_ isotopic ratios obtained from the reaction of laser‐ablated iridium atoms co‐deposited with pure dinitrogen at 4–5 K.

^14^N_2_	^15^N_2_	^14^N_2_ and ^15^N_2_	^14^N_2_/^15^N_2_ ratio	Assignment
2327.9	2249.9	2327.9, 2249.9	1.0347	N_2_ (perturbed)
2271.3	2195.2	[a]	1.0347	Ir(N_2_)^+^
2242.8	2168.2	[b]	1.0344	Ir_x_(N_2_)_*y*_
2221.7	2147.6	[b]	1.0345	Ir_x_(N_2_) _*y*_
2217.5	2143.5	[b]	1.0345	Ir_x_(N_2_)_*y*_
2214.3	2140.5	[b]	1.0345	Ir_x_(N_2_)_*y*_
2151.1	2079.5	2196.4, 2189.6 2151.1, 2101.8 2094.8, 2079.5	1.0344	Ir(NN)_2_
2150.0	2078.3	[a]	1.0345	Ir(NN)_2_ (site)
2003.2	1937.6	2003.3, 1992.8 1948.7, 1937.5	1.0339	N_3_^‐^
1955.8	1890.7	1955.8, 1912.4 1890.7	1.0344	Ir(N_2_)_2_^‐^
1657.5	1603.3	1657.6, 1649.2 1612.9, 1603.3	1.0338	N_3_
1652.4	1597.6	[a]	1.0343	N_3_ (site)
1102.8	1066.9	[a]	1.0336	IrN
1002.2	970.7	1002.2, 970.7	1.0325	IrIrN
857.1	831.2	857.1, 842.6 831.2	1.0312	Ir(N)_2_
780.2	755.2	780.2, 767.7 755.2	1.0331	IrNNIr

[a] Too weak. [b] Fall into congested area of the spectrum.

**Figure 6:**
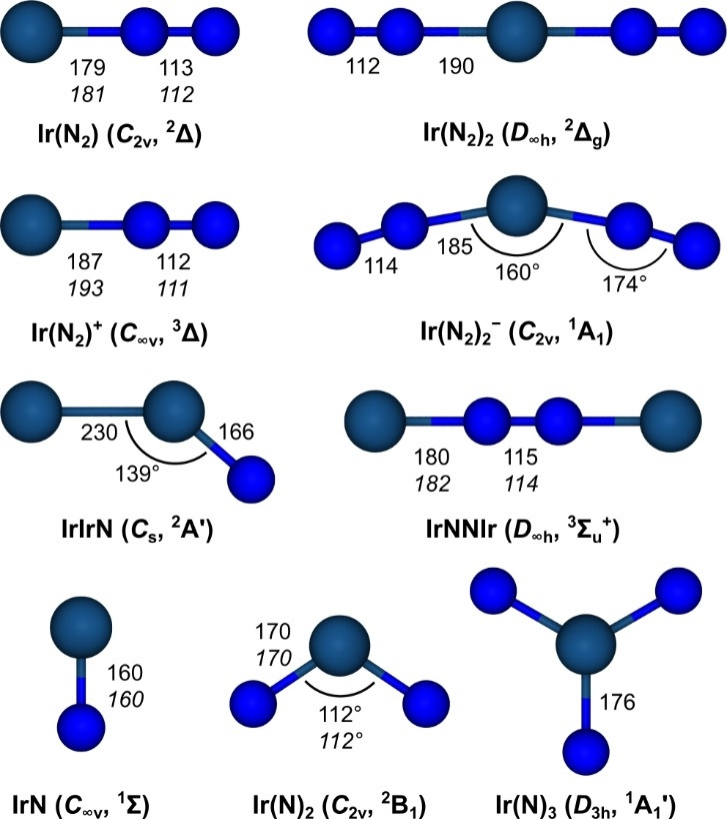
Structures, point group symmetries and electronic ground states calculated and optimized at the BP86/def2‐QZVP (regular font) and CCSD(T)/aVTZ(‐PP) (italic font) levels of theory.

Complementary spectra were also recorded in solid argon (Figures S1 and S2), and in the FIR region using neon as matrix host (Figure S3). A full list of absorptions found in argon matrices are given in Table 3. The bands centered at 2144.7, 2110.6, 2087.6, 1004.1, 848.2 cm^−1^ were assigned and marked in the Figures S1 and S2 to Ir(N_2_)_2_, Ir_x_(N_2_), Ir(N_2_), IrIrN and Ir(N)_2_, respectively. Bands obtained in the FIR region are shown in Figure S3. They are due to the three ^14/15^N isotopologues of Ir(N_2_)_2_ embedded in solid neon and located at 402.8, 397.4 and 393.1 cm^−1^, respectively. Optimized structures of the above‐mentioned dinitrogen and nitrido complexes of iridium were obtained at the DFT and CCSD(T) levels of theory and depicted in Figure 6. Computed harmonic frequencies of the reaction products are summarized in Table S1, and computed reaction enthalpies related to the formation of the observed reaction products are listed in Table 4. In the following the infrared spectra and the annealing and photolysis behavior of the reaction products, as well as our computational results are discussed, starting with the dinitrogen iridium complexes.

**Table 3:** Infrared absorptions (cm^−1^) and ^14^N_2_/^15^N_2_ isotopic ratios obtained from the reaction of laser‐ablated iridium atoms co‐deposited with dinitrogen diluted in argon at 4–5 K.

^14^N_2_	^15^N_2_	^14^N_2_ and ^15^N_2_	^14^N_2_/^15^N_2_ ratio	Assignment
2327.1	2249.2		1.0346	N_2_ (perturbed)
2144.7	2073.7	2187.5, 2144.7 2092.6, 2073.7	1.0342	Ir(N_2_)_2_
2138.5	2138.5			CO
2110.6	2040.1	2110.6, 2040.1	1.0346	Ir_*x*_(N_2_)
2087.6	2018.2	2018.2	1.0344	Ir(N_2_)
1004.1	972.7	1004.1, 972.7	1.0323	IrIrN
848.2				Ir(N)_2_

**Table 4:** Selected reaction enthalpies for the formation of iridium nitrogen compounds calculated at the DFT BP86/def2‐QZVP level of theory.

Reaction		Δ*H* [kJ mol^−1^]
Ir+N_2_	→Ir(N_2_)	−168
Ir(N_2_)+N_2_	→Ir(N_2_)_2_	−174
Ir(N_2_)_2_+N_2_	→Ir(N_2_)_3_	−32
Ir(N_2_)_3_+N_2_	→Ir(N_2_)_4_	−15
2 IrN	→IrNNIr	−96
Ir(N_2_)+Ir	→IrNNIr	−169
Ir+N	→IrN	−612
IrN+N	→Ir(N)_2_	−350
Ir(N)_2_+N	→Ir(N)_3_	−243
Ir(N)_2_	→Ir+N_2_	−20
Ir(N)_3_	→IrN+N_2_	−388
2 Ir	→IrIr	−422
IrIr+N	→IrIrN	−508
Ir(N)_3_	→Ir(N)_3_ TS	44^[a]^/41^[b]^
Ir(N)_3_ TS	→Ir(N)(N_2_)	−460^[a]^
Ir(N)_2_	→Ir(N)_2_ TS	244^[a]^/247^[b]^
Ir(N)_2_ TS	→Ir(N_2_)	−375^[a]^

[a] BP86/ZORA‐def2‐TZVPP(N)/SARC‐ZORA‐TZVPP(Ir). [b] M06‐L/ZORA‐def2‐TZVPP(N)/SARC‐ZORA‐TZVPP(Ir).

### Ir(N_2_)

The band observed at 2097.4 cm^−1^ in neon doped with 0.5 % ^14^N_2_ with a weaker matrix site at 2099.8 cm^−1^ is assigned to the Ir(N_2_) complex (Figure 1). This band is unaffected by broadband irradiation and grows upon annealing, while the sharp matrix site at 2099.8 cm^−1^ overtakes the initially stronger band at 2097.4 cm^−1^. The ^15^N counterpart exhibits the same behavior upon irradiation and is located at 2027.6 and 2030.0 cm^−1^, giving an isotopic frequency ratio of 1.0344 typical for N−N stretching modes. In the mixed ^14^N_2_ and ^15^N_2_ isotopic experiment the band at 2097.4 cm^−1^ is interfered by a stronger band associated with Ir(^14^N_2_)(^15^N_2_). However, due to the sharp, distinctive band shape of the matrix site at 2099.8 cm^−1^ after annealing, the weaker Ir(N_2_) band clearly stands out (Figure 1 e). The spectrum does not show any band related to a scrambled ^14^N/^15^N species in the mixed ^14^N_2_+^15^N_2_ experiment, thus, the characteristic doublet isotope pattern indicates a carrier bearing a single N_2_ unit. The corresponding absorption in the argon matrix is red‐shifted by 9.8 cm^−1^ relative to neon and the same isotopic ratio is found (Figure S1). Due to the formation of the higher coordinated species Ir(N_2_)_2_, the intensity of the Ir(N_2_) absorption band decreases with increasing amount of N_2_ and is absent in the neat dinitrogen spectrum. The assignments are supported by harmonic frequency calculations at the DFT and CCSD(T) level of theory. In analogy with RhNN, DFT and CCSD(T) calculations on Ir(N_2_) result in a linear *C*_∞v_ point group symmetry (Figure 6) for the ^2^Δ electronic ground state bearing an unpaired electron in an iridium centered degenerate δ molecular orbital, originating from d${}_{x{}^{2}-y{}^{2}}$ and d_*xy*_ iridium atomic orbitals. The lowest quartet state (^4^A′) is 104 kJ mol^−1^ higher in energy (Table S1). The deviations between the experimental N_2_ band position of Ir(N_2_) embedded in Ne and the computed values are 22 and 43 cm^−1^ for DFT and CCSD(T), respectively. The larger deviation of the superior CCSD(T) method is due to the fact that no harmonic contributions are considered, while the apparently better DFT value benefits from fortunate error cancelation. The NN stretching isotopic ratios $\tilde{\nu }$ (^14^N^14^N)/$\tilde{\nu }$ (^15^N^15^N) obtained by both, the DFT and CCSD(T) levels of theory are 1.0350, which is in good agreement with the experimental value of 1.0343.

### Ir(N_2_)^+^

The N−N stretching band of the cationic species Ir(N_2_)^+^ appeared red‐shifted by 172 at 2270.3 cm^−1^ and is observed in all experiments using N_2_/Ne mixtures as well as in neat N_2_, in which the band is blue‐shifted by 1.0 cm^−1^ (Figure 1). Selective irradiations using LED light sources of *λ*=656, 455, 405 and 365 nm did not affect the absorption intensity. However, full arc irradiation depleted, and annealing of the dinitrogen matrix to 30 K, destroyed the band entirely. The N−N stretch of the Ir(^15^N_2_)^+^ isotopologue is located at 2194.8 cm^−1^, resulting in an isotopic ratio of 1.0344, typical for modes involving two nitrogen atoms. As for the neutral species, no additional bands could be assigned to this species in the 1:1 ^14^N_2_/^15^N_2_ mixed isotope experiment, implying the presence of a single dinitrogen unit. Computational results at the DFT and CCSD(T) levels of theory support the assignment further. The calculated harmonic frequencies are at 2212 and 2286 cm^−1^, respectively. For [Ir(N_2_)]^+^ a ^3^Δ electronic ground state was found with one electron removed from a non‐bonding σ type molecular orbital. Compared to the neutral species the electronic ground state of the cation was computed to be 848 kJ mol^−1^ higher in energy.

### Ir(N_2_)_2_

The band centered at 2154.0 cm^−1^ with a matrix site at 2158.0 cm^−1^ obtained in solid neon doped with 0.5 % ^14^N_2_ and shown in Figure 2 remained unaffected by annealing but decreased dramatically upon irradiation with LED light of *λ*=455 nm. Annealing after photolysis increased the intensity of the initially weaker matrix site at 2158.0 cm^−1^. The ^15^N counterparts at 2082.4 and 2086.1 cm^−1^ result in an isotopic ratio of 1.0344. Increasing the amount of N_2_ in the solid Ne matrices strongly increases the intensity of the band and it is red‐shifted by 4.0 cm^−1^ in neat N_2_ (Figure 2). The mixed ^14^N_2_ and ^15^N_2_ spectrum displays a characteristic pattern for linear Ir(N_2_)_2_ consisting of the three antisymmetric N−N stretching modes of (^14^N_2_)Ir(^14^N_2_), (^14^N_2_)Ir(^15^N_2_) and (^15^N_2_)Ir(^15^N_2_) at 2154.0, 2100.3 and 2082.4 cm^−1^, respectively. Additionally, the symmetric N−N stretching mode of the (^14^N_2_)Ir(^15^N_2_) species becomes IR active due to lower point group symmetry *C*_∞v_ and is found at 2194.6 cm^−1^. Figure 1 f shows the ^14^N/^15^N isotope pattern of Ir(N_2_)_2_ arising from a 1:1 mixture of ^14^N_2_ and ^14^N_2_ (10 % in Ne), shown in a difference spectrum obtained by subtracting the spectra after and prior to selective photolysis with LED light of *λ*=455 nm. In the FIR spectrum shown in Figure S3 a 1:2:1 triplet ^14/15^N_2_ isotope pattern of Ir(N_2_)_2_ was also observed at 402.8, 397.4 and 393.1 cm^−1^ originating from a 1:1 mixture of ^14^N_2_ and ^14^N_2_ in solid neon which results in an isotopic ratio of 1.0247. Comparing our assignments to the frequencies calculated at the DFT level of theory there is a very good agreement for the antisymmetric N−N stretching mode at 2149 cm^−1^ and an isotopic ratio of 1.0349. The symmetric N−N stretching mode in Ir(^14^N_2_)(^15^N_2_) is calculated to be centered at 2182 cm^−1^ and the position of the antisymmetric Ir−N stretching mode at 439 cm^−1^, leading to an isotopic ratio of 1.0267. In analogy to the Ir(N_2_) complex, DFT and CCSD(T) calculations on Ir(N_2_)_2_ find a ^2^Δ_g_ ground state (*D*_∞h_ point group symmetry) having an unpaired electron located in a degenerate δ_g_ molecular orbital. The HOMO→LUMO (1δ_g_→2π_u_) excitation gives rise to the lowest quartet state ^4^Π_u_, which is 246 kJ mol^−1^ higher in energy than the electronic ground state.

### Ir(N_2_)_2_^−^

A strong band observed at 1955.8 cm^−1^ in neat ^14^N_2_ shown in Figures 2 and S7 decreases completely on annealing and is unaffected by broadband irradiation. The corresponding absorption in solid neon doped with 10 % ^14^N_2_ at 1956.4 cm^−1^ lead to a related ^15^N isotopologue absorption at 1890.3 cm^−1^ (Figure S7). Together with a stronger band at 1912.9 and a weak band at 1988.1 cm^−1^ which appeared in the 1:1 ^14^N_2_/^15^N_2_ spectrum a ^14/15^N isotope pattern similar to that of Ir(N_2_)_2_ is observed. Therefore, the band is assigned to the anionic complex Ir(N_2_)_2_^−^. The assignment is supported by DFT calculations, which predict a ^1^A_1_ singlet ground state of *C*_2*v*_ point group symmetry and infrared absorptions at 1988, 1940 and 1921 cm^−1^ for the antisymmetric N−N stretching modes of Ir(^14^N_2_)(^14^N_2_)^−^, Ir(^14^N_2_)(^15^N_2_)^−^ and Ir(^15^N_2_)(^15^N_2_)^−^. The experimental and calculated isotopic ratios are 1.0344 (in neat N_2_), 1.0350 (in solid Ne) and 1.0349 (DFT calc.) and are in very good agreement for the less interacting neon matrix. For a bent structure of Ir(NN)_2_^−^ and unlike the case of a linear Ir(N_2_)_2_, the symmetric N−N stretching mode is IR active. However, this band is not observed in the experiment, probably because of its low intensity, which is calculated to be 50 times lower than that of the antisymmetric mode. In the Ir(^14^N_2_)(^15^N_2_)^−^ isotopologue the intensity ratio of the symmetric and antisymmetric modes change to 1:4, and hence, the symmetric N−N stretching combination can be observed at 2031 cm^−1^ in the neat 1:1 ^14^N_2_/^15^N_2_ spectrum. Compared to the neutral complex the anion Ir(NN)_2_^−^ is 224 kJ mol^−1^ lower in energy at the DFT level of theory, which corresponds to the adiabatic electron affinity of Ir(N_2_)_2_.

### IrNNIr

Laser‐ablated iridium atoms co‐deposited with 10 % ^14^N_2_ in neon give raise to a band at 786.5 cm^−1^ which is unaffected by annealing to 12 K and vanishes after 10 min of irradiation with *λ*=455 nm (Figure 4). The same response was observed for a band at 761.6 cm^−1^ under the same conditions using ^15^N_2_. The isotopic triplet observed at 786.5, 774.1 and 761.6 cm^−1^ in a 1:1 mixture of ^14^N_2_ and ^15^N_2_ indicates an Ir−N stretching mode involving two equivalent nitrogen atoms. The intensity pattern of 1:2:1 suggests the presence of the isotopologue containing both isotopes, ^14^ n and ^15^N Based on the very good agreement of the band positions, isotopic pattern and isotopic ratio of the antisymmetric Ir−N stretching mode obtained by our quantum‐chemical calculations, the band was assigned to IrNNIr. This dimer could probably be formed by an oxidative coupling of two IrN molecules (Equation (1):

(1) $$2\;\mathrm{IrN}\to \mathrm{IrNNIr}\;\;\;\;\;\Delta H=-96\;\mathrm{kJ}\;\mathrm{mol}{}^{-1}$$

The observed isotopic ratios in solid neon and solid dinitrogen are 1.0327 and 1.0331, which are in very good agreement with the calculated DFT value of 1.0324. The calculated absorptions are 782, 769 and 757 cm^−1^ for the Ir^14^N^14^NIr, Ir^14^N^15^NIr and Ir^15^N^15^NIr isotopologues, respectively. The electronic ground state is found to be a triplet ^3^Σ_u_^+^, with the two unpaired electrons located at each of the metal centers in degenerated molecular orbitals of d${}_{x{}^{2}-y{}^{2}}$ ‐ and d_*xy*_‐character, reminiscent of the Ir(N_2_) complex. The N−N stretching mode is IR inactive and calculated to be centered at 2081 cm^−1^ at the DFT level of theory.

### IrN

A very weak band at 1102.8 cm^−1^ which was not observed in the initially formed solid ^14^N_2_ deposit but grew in upon annealing to 35 K and was destroyed by broadband irradiation, returned on subsequent annealing to 35 K (Figure 4). The corresponding absorption in ^14^N_2_ doped neon is blue‐shifted to 1111.1 cm^−1^ and the ^15^N counterparts were found at 1076.4 and 1066.9 cm^−1^ in neon and ^15^N_2_, respectively, while no band due to a mixed ^14/15^N isotopologue occurred in experiments using a 1:1 mixture of ^14^N_2_ and ^15^N_2_ (Figure 5). The assignment of this band to IrN is supported by a previous Fourier transform emission spectroscopic study of Ram and Bernath,15a in which a ground‐state fundamental Ir‐N stretching frequency of 1113.6 cm^−1^ for ^193^Ir^14^N was reported, revealing reasonable matrix shifts of −2.5 and −10.8 cm^−1^ for neon and solid dinitrogen. For the sake of completeness, DFT and CCSD(T) calculations were carried out and the results are listed in Table S1. The annealing behavior of IrN suggests a temperature induced mobility of N radicals reacting with iridium atoms to IrN (Δ*H*=−612 kJ mol^−1^).

### IrIrN

An intense band at 1002.2 cm^−1^ in solid ^14^N_2_ matrices and their ^15^N counterparts at 970.7 cm^−1^ grow in upon annealing to 35 K and remains unaffected by broadband irradiation (Figure 4). The much weaker bands located at 1004.4 and 972.8 cm^−1^ in solid neon doped with 10 % N_2_ depicted in Figure 3 show the same behavior and in experiments using a ^14^N_2_/^15^N_2_ mixture no additional band due to a mixed ^14/15^N isotopologue occurred, suggesting an Ir−N stretching mode with a single nitrogen atom involved. With IrN already assigned to a band centered about 100 cm^−1^ blue‐shifted, the carrier of this unknown band could be Ir(N)(N_2_), a dinitrogen complex of IrN, or IrIrN. Calculations at the DFT level of theory predict a fairly strong N≡N stretching mode for Ir(N)(N_2_) at 2110 cm^−1^, much higher than the Ir≡N stretching mode at 1085 cm^−1^, however, no such N≡N band could be identified in the spectrum and hence, ruled out an assignment to Ir(N)(N_2_). On the other side, infrared absorptions computed at the DFT level of theory support the assignment of the band at 1004.4 in solid neon to IrIrN, with calculated harmonic frequencies of 1054 and 1021 cm^−1^ for IrIr^14^N and IrIr^15^N, respectively, accounting for an isotopic ratio of 1.0323, which is very close to the experimental ones of 1.0325 in solid dinitrogen and neon. In analogy to IrN, the observed behavior upon annealing suggests a formation by mobilizing nitrogen radicals which react with Ir_2_ units present in the matrix. This is further supported by a computed reaction enthalpy for the formation of IrIrN from N atoms and the iridium dimer obtained at the DFT level of theory of Δ*H*=−508 kJ mol^−1^ (Table 4). The electronic ground state of the bent IrIrN structure in the *C*_s_ point group symmetry is a doublet ^2^A′ state.

### Ir(N)_2_

A very weak band, located at 853.5 cm^−1^ in solid neon doped with 0.5 % ^14^N_2_ and unaffected by broadband irradiations, presents a doublet pattern in neon doped with 0.5 % of a 1:1 mixture of ^14^N_2_ and ^15^N_2_ at 853.5 and 827.7 cm^−1^ (Figure 3). The picture changes when pure dinitrogen is used as matrix host: besides a slight blue‐shift of these bands to 857.1 and 831.2 cm^−1^, an intermediate band at 842.6 cm^−1^ appears, yielding a triplet pattern with an intensity ratio of about 1:2:1 (Figure 5). The observed triplet pattern is consistent with the involvement of two equivalent nitrogen atoms in an antisymmetric Ir−N stretching mode, such as in iridium dinitride, Ir(N)_2_. This assignment is supported by the fact that the intermediate band belonging to the ^14^NIr^15^N isotopologue is red‐shifted 1.6 cm^−1^ from the center, indicating a coupling between the symmetric and the anti‐symmetric Ir−N stretching modes, which in the lower point group symmetry *C*_s_ have the same a′ symmetry. The different patterns observed in solid neon and pure dinitrogen matrices can be explained by different reaction mechanisms leading to Ir(N)_2_: While a direct insertion of iridium atoms into a dinitrogen bond is proposed in nitrogen doped neon mixtures (Equation (2a)a), the reaction of IrN with N atoms preferentially occurred in solid dinitrogen matrices (Equation (2b)b).

(2a) $$\mathrm{Ir}+N{}_{2}\to \mathrm{Ir}\left(N\right){}_{2}\;\;\;\;\;\;\Delta H=+20\;\mathrm{kJ}\;\mathrm{mol}{}^{-1}$$

(2b) $$\mathrm{IrN}+N\to \mathrm{Ir}\left(N\right){}_{2}\;\;\;\;\;\;\Delta H=-612\;\mathrm{kJ}\;\mathrm{mol}{}^{-1}$$

While the reaction enthalpy of the direct insertion is slightly positive on the DFT level of theory, the high temperature of laser ablated iridium atoms can overcome this barrier and cryogenic conditions prevent the spontaneous elimination of a N_2_ unit. In contrast to osmium, spontaneous insertion into the NN triple bond at cryogenic conditions is not observed.^4^ Harmonic frequencies obtained by calculations on the DFT level of theory are in very good agreement, resulting in antisymmetric b_2_ stretching frequencies of 869, 853 and 842 cm^−1^ for Ir(^14^N)_2_, Ir(^14^N)(^15^N) and Ir(^15^N)_2_. The symmetric a_1_ Ir−^14^N stretching mode was not observed and calculated to be located at 1027 cm^−1^ having an intensity less than 4 % of the antisymmetric one. From the band positions of the antisymmetric stretching modes in the isotope substitution experiment an estimate of the upper limit of the N‐Ir‐N bond angle can be estimated to 130°,^34^ which is in agreement with the calculated angle of 112° for the *C*_2*v*_ (^2^B_1_) electronic ground state geometry.

### Ir(N)_3_

No band could be assigned to iridium trinitride, although we observed nitrogen atom mobility in the formation of Ir(N)_2,_ and the third addition of a nitrogen atom was calculated to be exothermic (Δ*H*=−243 kJ mol^−1^, Table 4). However, Table S1 shows that the integrated intensity of the IR active Ir−N stretching absorption in the observable region, the degenerate e′ mode, is calculated to be 1.4 km mol^−1^, which is about 3 % of the calculated integrated intensity of the corresponding very weak band assigned to Ir(N)_2_. The amount of iridium trinitride formed according to that mechanism would certainly be very low.

### Bonding considerations

#### Dinitrogen complexes of iridium

The nature of the metal nitrogen bond in selected product molecules and in Ir(N)_3_ will be discussed in terms of the relevant vibrational stretching modes as well as by analysis of the wavefunctions obtained at the BP86/def2‐QZVP level of theory. The coordination chemistry of the dinitrogen molecule is limited because it is a comparatively poor σ‐donor, weak π‐acceptor and its lack of dipole moment.^35^ The π‐donation of the iridium center into the π* molecular orbitals of the dinitrogen unit results in a weakening, or activation of the dinitrogen triple bond. The weakening of the N−N bond in dinitrogen complexes can be quantified experimentally by the red‐shift of the N−N stretching mode in the IR spectrum, comparing the N−N bond distances and, theoretically, by extracting information from the wavefunction. Several neutral PGM dinitrogen complexes have previously been studied by matrix isolation spectroscopy.^4^, ^5^, ^6^, ^7^, ^8^ Their experimental N−N stretching frequencies embedded in argon are given in Table S2 and provide a solid basis for discussing the nature of bonding in such homoleptic dinitrogen complexes. The red‐shift of the N−N stretching mode of Ir(N_2_) relative to that in free dinitrogen (2327.1 cm^−1^, Table 3) is 240.3 cm^−1^, which is less than the one for the group 8 metal dinitride Os(N_2_) and greater than that for the group 10 analogue Pt(N_2_). This trend is consistent with a decreasing ability of late transition metals to donate electron density into the π* orbitals of the coordinated N_2_ moiety due to less MO overlap caused by larger bond distances and decreasing d‐orbital energies. The same trend is observed with the corresponding first row transition metals.1a

The electron density at the bond critical point (*ρ*_b_) in a molecule can be taken as measure of the character of a bond and its bond order.^36^ The data presented in Table S3 shows a significant decrease of *ρ*_b_(NN) going from Ir(N_2_) (0.622) over Ir(N_2_)_2_ (0.592) down to IrNNIr (0.579), indicating a weakening of the corresponding N−N bond of the dinitrogen ligand within this series. This is also evident in the minimum structures shown in Figure 6, where the longest N−N bond lengths within this series of 115 pm is exhibited in the binuclear complex IrNNIr. In contrast to the electron density at the bond critical point (*ρ*_b_), which seems to be mainly affected by σ donation from the N_2_ ligand to the iridium center, the slightly longer N−N bond length in Ir(N_2_) (113 pm) compared to Ir(N_2_)_2_ (112 pm), is consistent with an increasing experimental N−N stretching frequency from Ir(N_2_) (2087.6 cm^−1^) to Ir(N_2_)_2_ (2144.7 cm^−1^), and can most likely be rationalized by a stronger π backdonation from the iridium center to the N_2_ ligand bonding in the Ir(N_2_) complex. Weakly activated N−N bond lengths are typically less than 112 pm,^35^ placing Ir(N_2_) and Ir(N_2_)_2_ at the upper end of the scale for what is considered weakly activated. The slightly stronger N_2_ activation in Ir(N_2_) compared to Ir(N_2_)_2_ is also supported by an NBO analysis, which results in NPA bond orders for the N−N bonds in Ir(N_2_), Ir(N_2_)_2_, and IrNNIr of 2.56, 2.64 and 2.51, respectively, as well as by the shorter calculated Ir−N bond distance in Ir(N_2_) of 179 pm compared to 190 pm in Ir(N_2_)_2_ (Table S3).

Comparing the N−N stretching modes of the ions [Ir(N_2_)]^+^ and [Ir(N_2_)_2_]^−^ with those of their neutral counterparts, a blue‐shift for the cation and a red‐shift for the anion is observed, which is consistent with the calculated changes in the corresponding N−N bond lengths (Table S3, Figure 6) and with the notion that oxidation of the metal center leads to a lower ability of π‐back‐donation, while reduction leads to an increase.2c In both cases, the addition or subtraction of an electron does not change the occupation number of the π‐system, but leads to an oxidation or reduction of the iridium center. Compared to a shift for the C−O stretching frequency in Ir(CO)^+^ and Ir(CO)_2_^−^ with respect to neutral Ir(CO) of +132 and −29 cm^−1^, respectively,^37^ the frequency shift for the isoelectronic dinitrogen complexes is with +170 and −198 cm^−1^ significantly larger. The higher sensitivity of the N−N stretching frequency upon oxidation or reduction of the metal center compared to the C−O frequency is another indication for the importance of π‐back‐bonding as the most significant contribution to the Ir−N bond strength.2c On the other side, the red‐shift of the N−N and C−O stretching frequencies in the neutral Ir(N_2_) and Ir(CO) complexes with respect to the free ligands is with 170 cm^−1^ (9.8 %) higher in Ir(N_2_) compared to 132 cm^−1^ (5.4 %) in Ir(CO). As pointed out by Pelikán and Boča,2c the larger red‐shift for the N−N stretch does not indicate a stronger π‐back‐donation in the Ir(N_2_) complex, since both interactions, σ‐donation and π‐acceptance lead to a weakening of the N−N bond, while in the Ir(CO) complex σ‐donation leads to an increase and π back‐donation to a decrease in the C−O bond strength. Taking the better σ‐donor ability of CO compared to NN into account,^38^ CO must be considered a stronger π‐acceptor than the N_2_ ligand.

**Figure 7:**
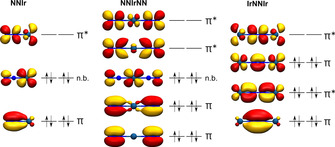
Ir(N_2_), IrNNIr and Ir(N_2_)_2_ Kohn–Sham molecular orbitals of their π‐system with an iso surface value of 0.04 Å^−1^ obtained at the BP86/def2‐QZVP level of theory.

In Figure 7 the frontier molecular orbitals of the π‐system are shown for the neutral, linear dinitrogen complexes Ir(N_2_), Ir(N_2_)_2_, and IrNNIr. Each of them is comprised of the degenerate 2p_*y*_ and 2p_*z*_ atomic orbitals of the nitrogen atoms and the 3d_*xy*_ and 3d_*xz*_ atomic orbitals of the iridium atoms involved. For IrNNIr with 12 π‐electrons the first three of four pairs of the molecular π‐orbitals are fully occupied (Figure 7, right). The first pair essentially forms the π‐bonds of the NN unit, the second pair contains the corresponding N−N anti‐bonding molecular orbitals of the first set, and the third pair of the π‐bonding orbitals are Ir−N anti‐bonding and N−N bonding. The electronic ground state of ^3^Σ_u_^+^ arises from two unpaired electrons residing in non‐bonding molecular orbitals, essentially formed by the non‐bonding iridium 3d_*yz*_ atomic orbitals (not shown in Figure 7). From isotopic triplet observed in a 1:1 mixture of ^14^N_2_ and ^15^N_2_ for the Ir−N stretching vibration in the IR spectrum it has been concluded that IrNNIr is likely formed during matrix deposition by the coupling of two IrN units. A very similar behavior was reported for the [N_2_{Ir(PNP)}_2_] (PNP=N(CHCHP*t*Bu_2_)_2_) pincer complex, holding the same 12 electron IrNNIr π‐system, which was observed to be formed in solution at room temperature by coupling of two terminal [IrN(PNP)] nitrido complexes.1c This coupling reaction can be viewed as the reverse of splitting a bridging dinitrogen ligand into separate nitrido complexes, which recently was investigated for [NIr(PNP)]_2_^n+^ (*n*=0, 1, 2).^39^ For these complexes reaction enthalpies of the coupling reaction of 2[NIr(PNP)]^n+^, with 2*n*=0, 1, and 2, are exotherm and calculated to Δ*H=−*510, −425 and −382 kJ mol^−1^ (D3BJ‐PBE0(Cosmo (THF))/def2‐TZVP//D3BJ‐PBE0/def2‐SVP) respectively.^39^ In contrast to these results coupling of two IrN complexes, bare of any additional ligands and under solvent‐free conditions in an argon matrix, is significantly less exothermic with Δ*H*=−96 kJ mol^−1^. The lower reaction enthalpy for the latter coupling reaction can be explained by the formation of two strong triple bonds in the Ir≡N units.

#### Nitrido complexes of iridium

The diatomic iridium nitride, IrN, was investigated extensively using emission^14^, 15a, 15b and optical Zeeman15e spectroscopy as well as high level ab initio methods.15b It has a closed shell ^1^Σ^+^ ground state comprising an Ir≡N triple bond. Our AIM analysis assign charges of 0.278 to iridium and −0.278 to nitrogen (Table S3), while NBO analysis shows NPA charges of −0.032 and 0.032 for iridium and nitrogen, respectively, and an NPA bond order of 2.82. The NPA charge close to zero and the low AIM charges imply that the bond nature is mostly covalent.

**Scheme 1:**
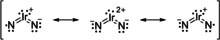
Lewis structure for the homoleptic diinitrido iridium.

The calculated Ir−N bond lengths of iridium dinitride, Ir(N)_2_, in its ^2^B_1_ electronic ground state of 170 pm is considerably larger than the expected ones for a triple bond judged on the calculated bond lengths in iridium mononitride of 160 pm, which also corresponds well to the sum of the triple‐bond covalent radii of iridium and nitrogen of 160 pm.^40^ From the sum of reported double‐41 and triple‐bond covalent radii of iridium and nitrogen of 160 and 175 pm, respectively, the bond order in Ir(N)_2_ can be estimated to be between a triple and a double bond, while the computed NPA bond order is 2.06. The NPA (AIM) charges are significantly higher compared to diatomic Ir≡N, amounting to 0.588 (0.896) and −0.294 (−0.448) for iridium and nitrogen, respectively. The higher charges and lower covalent bond order of the dinitride compared to the mononitride suggest an Ir=N double bond in the former one, which is shortened due to a higher ionic character. The valence molecular orbitals and occupation numbers obtained from SA‐CASSCF(15,12)/cc‐pVTZ(‐PP) calculations (for details see Computational Details) for the ^2^B_1_ electronic ground state are depicted in Figure S4. These calculations reveal a lone pair (5a_1_) at the iridium center, two σ‐bonding orbitals (6a_1_ and 4b_2_), two π‐bonding orbitals (2b_1_ and 1a_2_), and their anti‐bonding σ* (8a_1_ and 6b_2_) and π* counterparts (3b_1_ and 2a_2_). We note that one unpaired electron resides in the 3b_1_ π* orbital. Additionally, two essentially doubly occupied non‐bonding orbitals (7a_1_ and 5b_2_) remain at the nitrogen ligands, and finally, there is a high‐lying, low‐occupied non‐bonding orbital (9a_1_) with contributions from the Ir(6s), Ir(5d${}_{x{}^{2}-y{}^{2}}$ ), Ir(6p_*z*_) and N(2p_*z*_) atomic orbitals (Figure S4). An effective bond order (EBO) of 1.56 can be estimated for the Ir−N bond by counting the occupation numbers of the bonding‐ and anti‐bonding molecular orbitals, neglecting the slightly bonding characters of the nonbonding orbitals 7a_1_ and 5b_2_. For the ^2^B_1_ electronic ground state the presence of nitrogen‐centered unpaired electrons can be ruled out, however, an estimation of low‐lying electronic states using SA‐CASSCF(15,12)/cc‐pVTZ(‐PP) calculations show that the lowest lying quartet state is 84 kJ mol^−1^ and the lowest lying sextet state 252 kJ mol^−1^ higher in energy than the electronic ground state (Figure S5 and Table S4). The most dominant configuration of the electronic ground state is a_1_^6^ b_1_^3^ b_2_^4^ a_2_^2^ with a weight of 0.86 (74 %). Other contributions are small and distributed over the whole expansion space. This electron configuration can be described by the resonance Lewis structure shown in Scheme 1, in which an integral formal oxidation state cannot be assigned to the iridium center a priori.

If we resort to MO theory and wavefunction analysis, spin populations can give insight into which extend the unpaired electron is localized either on iridium, or at the nitrido ligands. Mulliken and Loewdin population analysis yield spin populations of 0.40 and 0.47 at the iridium center, which means the ligands do not allow the definition of a clear‐cut integral oxidation state. The formal oxidation state lies between +V and +VI, with slightly more weight on the side of +V. The nitrido ligands must be considered as non‐innocent.^42^

**Scheme 2:**
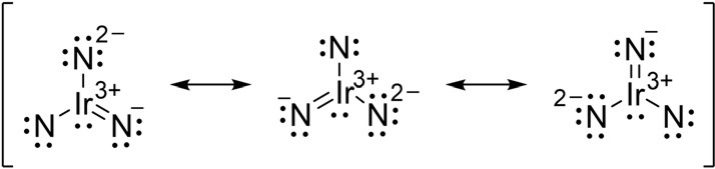
Lewis resonance structure for the homoleptic trinitrido iridium(VII) complex Ir(N)_3_.

Iridium trinitride is an intriguing compound since iridium would formally be considered in the oxidation state +IX, which so far was experimentally realized only for the cation [IrO_4_]^+^, and more recently claimed for the experimentally unknown nitrido compound NIrO_3_. Another candidate for the oxidation state +IX could be Ir(N)_3_, provided that all 5d electrons from the valence shell of iridium can be formally assigned to the nitrogen ligands and no lone pair remains on the iridium atom. We have investigated Ir(N)_3_ at the BP86/def2‐QZVP level of theory and found a regular *D*_3*h*_ structure with an Ir−N bond length of 176 pm in the ^1^A_1_′ ground electronic state (Figure 6). We have further analyzed the occupied molecular orbitals at the R‐BP86/ZORA‐def2‐TZVPP(N)/SARC‐ZORA‐TZVPP(Ir) level of theory and depicted the valence molecular orbitals in Figure S6. These calculation reveals a degenerate pair of σ‐bonding orbitals (4e′) as well as a degenerate pair of π‐bonding orbitals (1e“). In addition, seven ligand‐centered lone pairs (4a_1_′, 2a_2_′′, 2×5e′, MO's arising from the N(2s) orbitals are not shown in Figure S6) can be assigned and a metal centered d‐orbital is attributed to the highest occupied MO (HOMO, 5a_1_′). We note that the lowest unoccupied MO (LUMO, 1a_2_′) is a nonbonding ligand‐centered MO. Thus, our analysis shows that the nitrido ligands in Ir(N)_3_ behave as non‐innocent ligands as well,^42^ meaning that an essentially non‐bonding iridium (d${}_{z{}^{2}}$ ) orbital (5a_1_′) is filled by two electrons at the expense of a ligand delocalized nonbonding LUMO (1a_2_′). This bonding situation, which is consistent with a formal oxidation state of +VII rather than +IX for the iridium atom in Ir(N)_3_, can be approximately described by the resonance Lewis structures shown in Scheme 2.

This electronic description is supported by the calculated AIM and NPA charges shown in Table S3. While the nitrogen atoms in Ir(N)_3_ adopt a charge, which is very close to the one found in Ir(N)_2_, the charge at the iridium center raised to about 3/2 of those in Ir(N)_2_.

**Figure 8:**
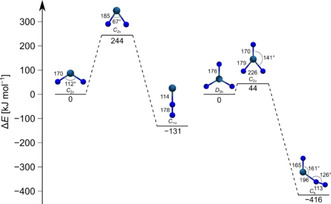
Stationary points on the doublet (singlet) potential energy surface of the decomposition pathways of Ir(N)_2_ (Ir(N)_3_) leading to elimination and complexation of N_2_ calculated at the BP86/ZORA‐def2‐TZVPP(N)/SARC‐ZORA‐TZVPP(Ir) level of theory.

The most favored decomposition pathway for doublet Ir(N)_2_ and singlet Ir(N)_3_ is found to be the elimination of dinitrogen, which is exothermic by Δ*H*=−20 and −388 kJ mol^−1^ for Ir(N)_2_ and Ir(N)_3_, respectively. According to our all‐electron R‐BP86/ZORA‐def2‐TZVPP(N)/SARC‐ZORA‐TZVPP(Ir) calculation the lowest energy pathway for dinitrogen elimination proceed by cleavage of an Ir−N bond and formation a dinitrogen complex (Figure 8). The nitrido complexes are separated from their dinitrogen coordinated isomers by a barrier of 244 and 44 kJ mol^−1^ for Ir(N)_2_ and Ir(N)_3_, respectively. The corresponding transition states on the quartet and triplet surfaces of Ir(N)_2_ and Ir(N)_3_, respectively, have also been investigated, and found to be higher in energy with 257 and 114 kJ mol^−1^ above the respective minimum structures. According to these results the kinetic stability with respect to dinitrogen elimination of Ir(N)_3_ is rather low, while Ir(N)_2_ is kinetically stable and the isomeric dinitrogen complex Ir(N_2_) has indeed been detected in the present study.

## Conclusions

Laser‐ablated iridium atoms were allowed to react with dinitrogen and nitrogen atoms formed from N_2_ molecules by plasma radiation and the products were isolated in solid neon, argon and nitrogen matrices and identified by their infrared spectra. The assignments are supported by ab initio and first principle calculations as well as ^14/15^N isotope substitution experiments. The neutral and ionic iridium dinitrogen complexes Ir(N_2_), Ir(N_2_)^+^, Ir(N_2_)_2_, Ir(N_2_)_2_^−^, IrNNIr were formed and assigned by their characteristic *N*‐N stretching frequencies at 2097.4, 2270.3, 2154.0, 1956.4 and 786.5 cm^−1^, respectively. In addition, the nitrido complexes IrN, Ir(N)_2_ and IrIrN were observed and assigned to Ir−N stretching bands centered at 1111.1, 853.5 and 1004.4 cm^−1^, respectively. While Ir(N)_2_ can be formed by a photo‐rearrangement of the corresponding dinitrogen complex Ir(N_2_) or from N atoms and IrN, the latter process was deduced from ^14/15^N isotopic experiments. The threefold coordinated iridium trinitride complex Ir(N)_3_ was not be observed. The structural and electronic properties of the dinitrogen ligand in the N_2_ complexes are discussed with respect to dinitrogen activation upon complexation. The largest dinitrogen activation was observed in the neutral, linear binuclear IrNNIr complex and in the anionic Ir(N_2_)_2_^−^. Also, the electronic structures of the nitrido complexes Ir(N)_2_ and Ir(N)_3_ were investigated by DFT and ab initio calculations. The dinitride Ir(N)_2_ adopts a bent structure in a ^2^B_1_ electronic ground state with one unpaired electron in a delocalized π* molecular orbital (3b_1_) and an additional lone pair on the iridium center. Ir(N)_3_ has a *D*_3*h*_ structure in the lowest energy electronic state in which a lone pair can be attributed to a nonbonding iridium centered 5d_z_2 orbital (5a_1_′) and a formal oxidation state for iridium of +VII rather than +IX can be deduced. The lowest energy decomposition pathway of these nitrido complexes has been found computationally to proceed via a rearrangement to the isomeric dinitrogen complexes.

## Conflict of interest

The authors declare no conflict of interest.

## Supporting information

As a service to our authors and readers, this journal provides supporting information supplied by the authors. Such materials are peer reviewed and may be re‐organized for online delivery, but are not copy‐edited or typeset. Technical support issues arising from supporting information (other than missing files) should be addressed to the authors.

Supplementary
