# Supplementary material for: A Cornucopia of Iridium Nitrogen Compounds Produced from Laser‐Ablated Iridium Atoms and Dinitrogen
Source: Chemistry. 2020 Apr 30;26(33):7384–94. doi: 10.1002/chem.201905514 (PMC7317413; doi:10.1002/chem.201905514)
Supplement: Supplementary file 1 — Supplementary [file CHEM-26-7384-s001.pdf]

Chemistry–A European Journal

Supporting Information

A Cornucopia of Iridium Nitrogen Compounds Produced from Laser-Ablated Iridium Atoms and Dinitrogen

Tony Stüker, Helmut Beckers, and Sebastian Riedel*[a]

A Cornucopia of Iridium Nitrogen Compounds Produced from Laser-Ablated Iridium Atoms and Dinitrogen

Table of Contents

Table of Contents 1

Supplemental Tables 3

Supplemental Figures 5

Supplemental computational results 10

BP86/def2-QZVP 10

BP86/ZORA-def2-TZVPP(N)/SARC-ZORA-TZVPP(Ir) 17

M06-L/ZORA-def2-TZVPP(N)/SARC-ZORA-TZVPP(Ir) 18

CCSD(T)/aug-cc-pVTZ(-PP) 19

References 22

Supplemental Tables

Table S1. Low-energy electronic state configuration (sorted by increasing energy) and vibrational frequencies (in cm^{-1}) of selected nitrogen containing molecules.

Molecule	Point Group	Electronic state and HOMOs		ΔE	Vibrational frequencies (Intensities) [Symmetry] $\tilde{\nu}^c$
N_2	$D_{\infty h}$	$^1\Sigma^+$	($1\sigma_g^2\ 1\sigma_u^2\ 1\pi_g^4\ 2\sigma_g^2$)		2348 (0) [σ_g]
N_3		$^2\Pi_g$	($1\pi_u^4\ 3\sigma_u^2\ 4\sigma_g^2\ 1\pi_g^4$)	0	479 (9) [π_u], 1716 (142) [σ_u]
N_3^-		$^1\Sigma_g^+$	($1\pi_u^4\ 3\sigma_u^2\ 4\sigma_g^2\ 1\pi_g^4$)	−263	2042 (894) [σ_u]
IrN	$C_{\infty v}$	$^1\Sigma$	($5\sigma^2\ 2\pi^4\ 1\delta^4\ 6\sigma^2$)	0	1209 (36) [σ]
					1195 (38) [σ]
	$C_{\infty v}$	$^3\Pi$	($2\pi^4\ 1\delta^4\ 6\sigma^1\ 3\pi^1$)	+95	1039 (15) [σ]
$Ir(N)_2$	C_{2v}	$^2B_1$	($2b_1^2\ 6a_1^2\ 5b_2^2\ 7a_1^2\ 3b_1^1$)	0	869 (41) [b_2]
	C_{2v}	$^2B_2$	($2b_1^2\ 6a_1^2\ 7a_1^2\ 5b_2^1\ 3b_1^2$)	+103	1119 (595) [b_2]
		$^2A_1$	($2b_1^2\ 6a_1^2\ 5b_2^2\ 7a_1^1\ 3b_1^2$)	+111	1005 (3) [b_2], 990 (1) [a_1]
		$^4A_2$	($6a_1^2\ 7a_1^2\ 5b_2^1\ 3b_1^1\ 8a_1^1$)	+59	958 (11) [a_1], 466 (595) [b_2]
$Ir(N)_3$	D_{3h}	$^1A_1'$			968 (0) [a1'], 770 (2 × 0.7) [e']
IrN_2	$C_{\infty v}$	$^2\Delta$	($7\sigma^2\ 3\pi^4\ 1\delta^3\ 8\sigma^2$)	0	382 (3) [π], 382 (3) [π], 584 (10) [σ^+], 2119 (283) [σ^+]
					377 (3) [π], 377 (3) [π], 549 (29) [σ^+], 2140 (463) [σ^+]
	C_s	$^4A'$	($4a''^2\ 11a'^1\ 12a'^1\ 13a'^1$)	+104	143 (20) [a'] 332 (0) [a'], 2119 (312) [a']
$Ir(N_2)^-$	$C_{\infty v}$	$^1\Sigma^+$	($7\sigma^2\ 3\pi^4\ 1\delta^4\ 8\sigma^2$)	−182	429 (3) [π], 429 (3) [π], 654 (5) [σ^+], 1955 (675) [σ^+]
					434 (3) [π], 434 (3) [π], 648 (12) [σ^+], *1963 (1018)* [σ^+]
$Ir(N_2)^+$	$C_{\infty v}$	$^3\Delta$	($7\sigma^2\ 3\pi^4\ 1\delta^3\ 8\sigma^1$)	+848	341 (1) [π], 341 (1) [π], 488 (15), 2212 (48)
					313 (1) [π], 313 (1) [π], 401 (14) [σ^+], 2286 (24) [σ^+]
$Ir(N_2)_2$	$D_{\infty h}$	$^2\Delta_g$	($2\pi_g^4\ 7\sigma_g^2\ 1\delta_g^3$)	0	439 (99), 2149 (1001)
	$D_{\infty h}$	$^4\Pi_u$	($2\pi_g^4\ 1\delta_g^2\ 7\sigma_g^2\ 2\pi_u^1$)	+246	276 (30), 1964 (1793)
$[Ir(N_2)_2]^+$	$D_{\infty h}$	$^3\Delta_g$	($2\pi_g^4\ 7\sigma_g^2\ 1\delta_g^2$)	+846	383 (48) [σ_u], 2269 (173) [σ_u]
	$D_{\infty h}$	$^1\Sigma_g^+$	($2\pi_g^4\ 7\sigma_g^2\ 1\delta_g^2$)	+866	383 (48) [σ_u], 2239 (174) [σ_u]
	$D_{\infty h}$	$^1\Sigma_g^+$	($2\pi_g^4\ 7\sigma_g^0\ 1\delta_g^4$)	+944	379 (46) [σ_u], 2209 (211) [σ_u]
$[Ir(N_2)_2]^-$	C_{2v}	$^1A_1$	(($2a_2^2\ 8b_1^2$) $9a_1^2$ ($3b_2^2\ 10a_1^2$))	−224	486 (69) [b_1], 1988 (1926) [b_1], 2052 (38) [a_1]
$Ir(N_3)$	C_s	$^3A''$	($13a''^2\ 4a''^2\ 14a'^2\ 15a''^1\ 5a''^1$)		2020 (407) [a'], 1153 (53) [a']
IrNNIr	$D_{\infty h}$	$^3\Sigma_u^+$	$6\sigma_g^2\ 2\pi_u^4\ 1\delta_g^3\ 1\delta_u^3$		2081 (0) [σ_g], 781 (162) [σ_u]
					2103 (0) [σ_g], 729 (361) [σ_u]
$NIr(N_2)$	C_s	$^1A'$			2110 (388) [a'], 1085 (27) [a']
IrIrN	C_s	$^2A'$	($13a'^2\ 14a'^2\ 5a''^2\ 6a''^2\ 15a'^1$)		1054 (60) [a']

[c]Selected vibrational absorptions with intensities greater than 0 and wavenumbers in observable range. See detailed computational results for all data.

TableTable S2. Overview of IR bands (in cm^{-1}) of dinitrogen complexes of the platinum group metals obtained in solid argon matrices.

Metal	$M(^{14}N_2)$	$M(^{15}N_2)$	$\Delta\nu_{N_2}$ c)	$M(^{14}N_2)_2$	$M(^{15}N_2)_2$	$\Delta\nu_{N_2}$ c)	$M(^{14}N_2)_2^-$	$M(^{15}N_2)_2^-$	$\Delta\nu_{N_2}$ c)
Ru[1]	2034.6	1967.1	293	2077.6	2008.6	250			
Rh[2]	2153.3	2081.7	175	2185.9	2112.8	142	1958.9	1893.4	369
Pd[3]	2213.0	2138.7	115	2234.0	2159.5	94			
Os[1]	2044.2	1976.2	283	2083.2	2013.9	245			
Ir	2087.6	2018.2	240	2144.7	2073.7	183	1956.4[a]	1890.3[a]	371[a]
Pt[4]	2168.5	2096.2	159	2195.4	2122.2	133	1862.5[b]	1803.5[b]	465[b]

[a] Recorded in neon matrix. [b] Recorded in pure dinitrogen matrix. [c] Red shift relative to uncoordinated N_2.

Table S3. NPA- and QTAIM results, bond lengths of selected iridium nitrogen molecules obtained by analyzing the BP86/def2-QZVP wavefunctions..

Property		NN	$(NH_2)_2$	Ir(NN')	Ir(NN')$_2$	IrNNIr	$Ir(N_2)^+$	$Ir(N_2)_2^-$	IrN	$Ir(N)_2$	$Ir(N)_3$
Bond length	Ir-N	-	-	179	190	180	187	185	160	170	176
	N-N	110	144	113	112	115	112	114	-	-	-
QTAIM Charge	Ir	-	-	0.227	1.611	0.227	0.954	–0.018	0.278	0.896	1.250
	N	0.000	-0.667	–0.264	–0.499	–0.227	–0.191	–0.154	–0.278	–0.448	–0.417[a]
	N’	-	-	0.037	–0.307	-	0.237	–0.337	-	-	-
AIM ρ_b	Ir-N	-	-	0.209	0.111	0.205	0.171	0.178	0.378	0.290	0.260
	N-N	0.700	0.298	0.622	0.592	0.579	0.648	0.613	-	-	
NPA Charge	Ir	-	-	0.069	0.087	0.087	0.926	–0.46	–0.032	0.588	0.847
	N	0.000	-0.678	-0.032	–0.070	–0.087	–0.122	–0.08	0.032	–0.294	–0.282
	N’	-	-	-0.037	0.027		0.196	–0.19	-	-	-
NPA B.O	Ir-N	-	-	0.65	0.38/0.43	0.62	0.50	0.55	2.82	2.06	1.74[a]
	N-N	3.01	1.02	2.56	2.64	2.51	2.55	2.61	-	-	

[a]Averaged values.

Table S4. Absolute and relative energies of the two lowest electronic states of all irreproducible representation of doublet, quartet, and sextet spin states of molecular iridium dinitride $Ir(N)_2$.

	Doublet		Quartet		Sextet	
Irrep.	*E* (hartree)	Δ*E* (kJ mol^{-1})	*E* (hartree)	Δ*E* (kJ mol^{-1})	*E* (hartree)	Δ*E* (kJ mol^{-1})
A_1	–212.5839098	50	–212.5115669	240	–212.5067341	252
A_1	–212.5572517	120	–212.4780023	328	–212.4098773	507
B_1	–212.6029061	0	–212.5594627	114	–212.4358756	439
B_1	–212.5285217	195	–212.5050720	257	–212.4212219	477
B_2	–212.5565516	122	–212.5285079	195	–212.4148392	494
B_2	–212.5190630	220	–212.5074391	251	–212.3828112	578
A_2	–212.5479788	144	–212.5709677	84	–212.4102628	506
A_2	–212.5377015	171	–212.4960646	281	–212.3931535	551

Supplemental Figures

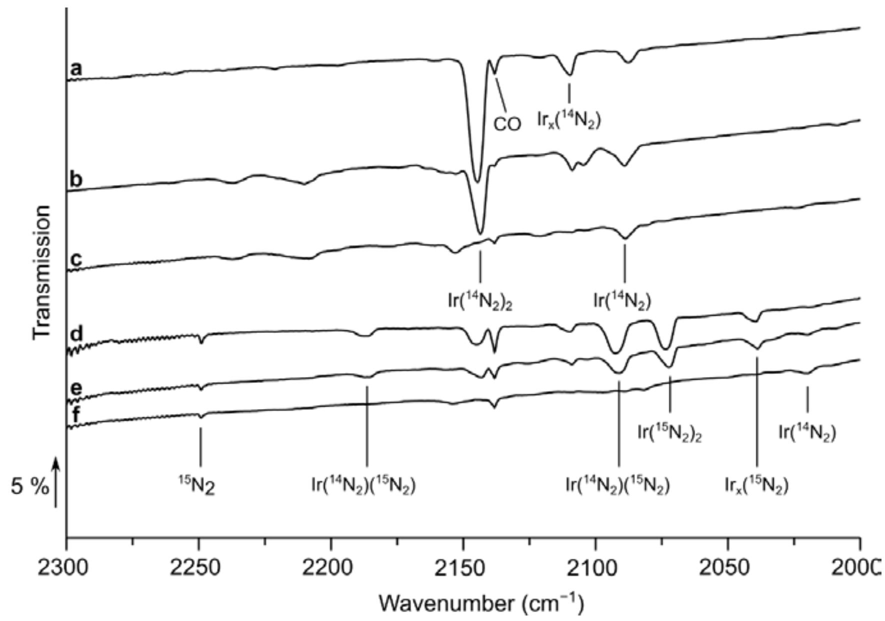

Figure S1. Infrared transmission spectra in the 2000–2350 cm^{-1} region of the reaction products of laser-ablated iridium atoms with 10 % $^{14}N_2$ (**a**, **b** and **c**) or a 1:1 mixture of $^{14}N_2$ and $^{15}N_2$ (**d**, **e** and **f**). Spectrum **a** and **d** were recorded after 90 min deposition, **b** and **e** after annealing to 25 K, and **c** and **f** were taken after irradiation with an LED light of 455 nm wavelength for 10 min.

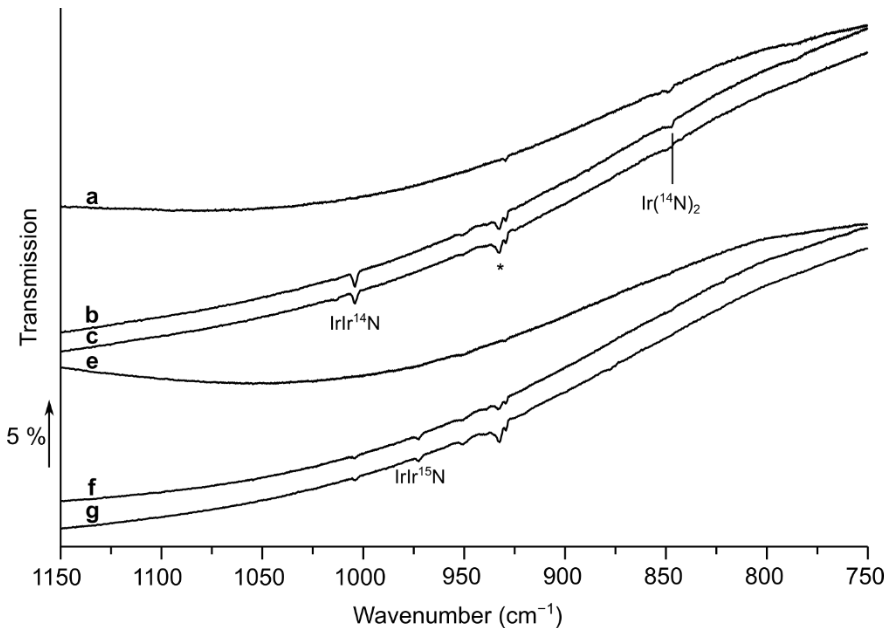

Figure S2. Infrared transmission spectra in the 750–1150 cm^{-1} region of the reaction products of laser-ablated iridium atoms with 10 % $^{14}N_2$ (**a**, **b** and **c**) or a 1:1 mixture of $^{14}N_2$ and $^{15}N_2$ (**d**, **e** and **f**). Spectrum **a** and **d** were recorded after 90 min deposition, **b** and **e** after annealing to 25 K, and **c** and **f** were taken after irradiation with an LED light of 455 nm wavelength for 10 min. Signal group highlighted with the asterisk belongs to nitrogen-less impurities which do not exhibit an isotopic shift.

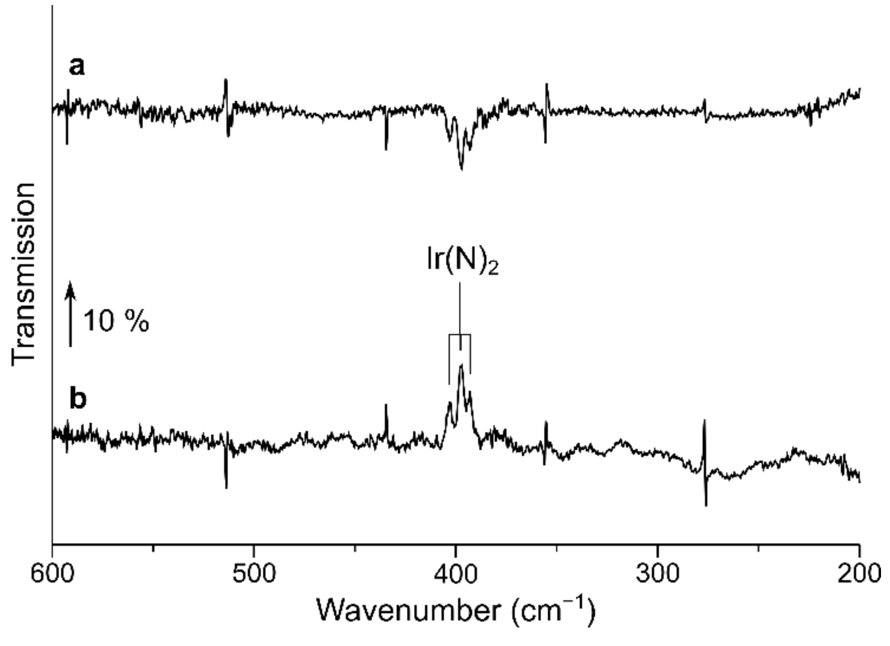

Figure S3. Infrared transmission spectrum in the 200–600 cm^{-1} region of the reaction products of laser-ablated iridium atoms with 10 % of a 1:1 mixture of $^{14}N_2$ and $^{15}N_2$ diluted in neon. Spectrum **a** was taken after 90 min of deposition, difference spectrum **b** was recorded after irradiating the matrix with LED light of 455 nm wavelength for 10 min.

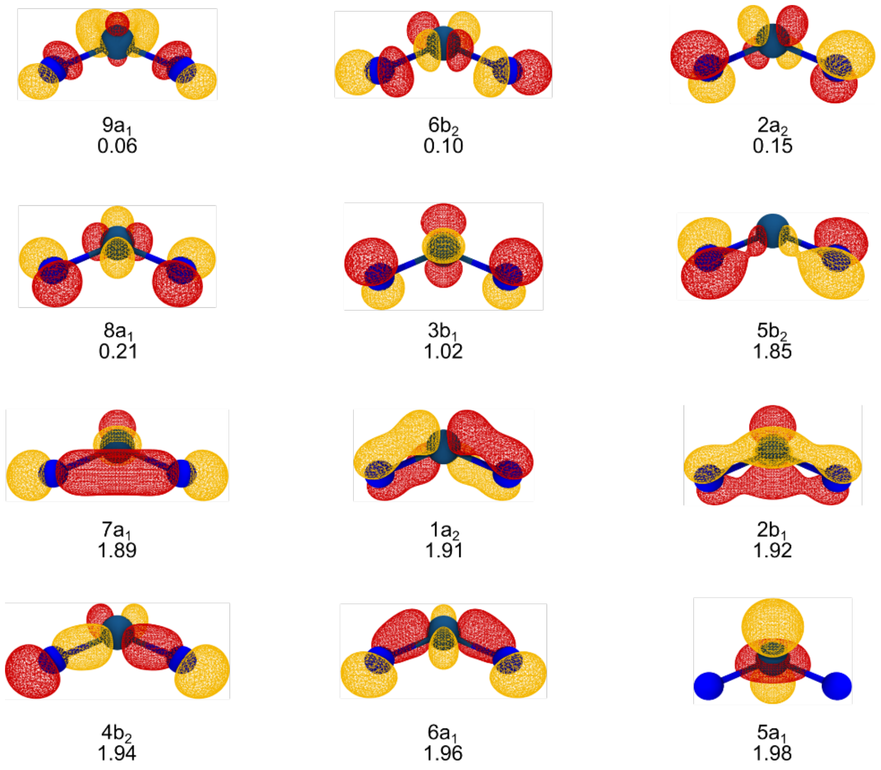

Figure S4. Valence natural molecular orbitals with an isosurface value of 0.04 Å^{-1} at the CASSCF(15,12)/cc-pVTZ(-PP) level of theory with occupation numbers and orbital wavefunction symmetry.

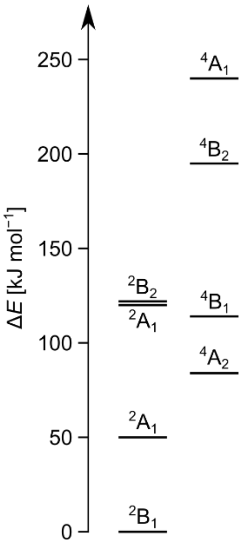

Figure S5. The four lowest electronic doublet and quartet states of $Ir(N)_2$ calculated using SA-CASSCF(15,12)/cc-pVTZ at the minimum geometry obtained at the BP86/def2-QZVP level of theory.

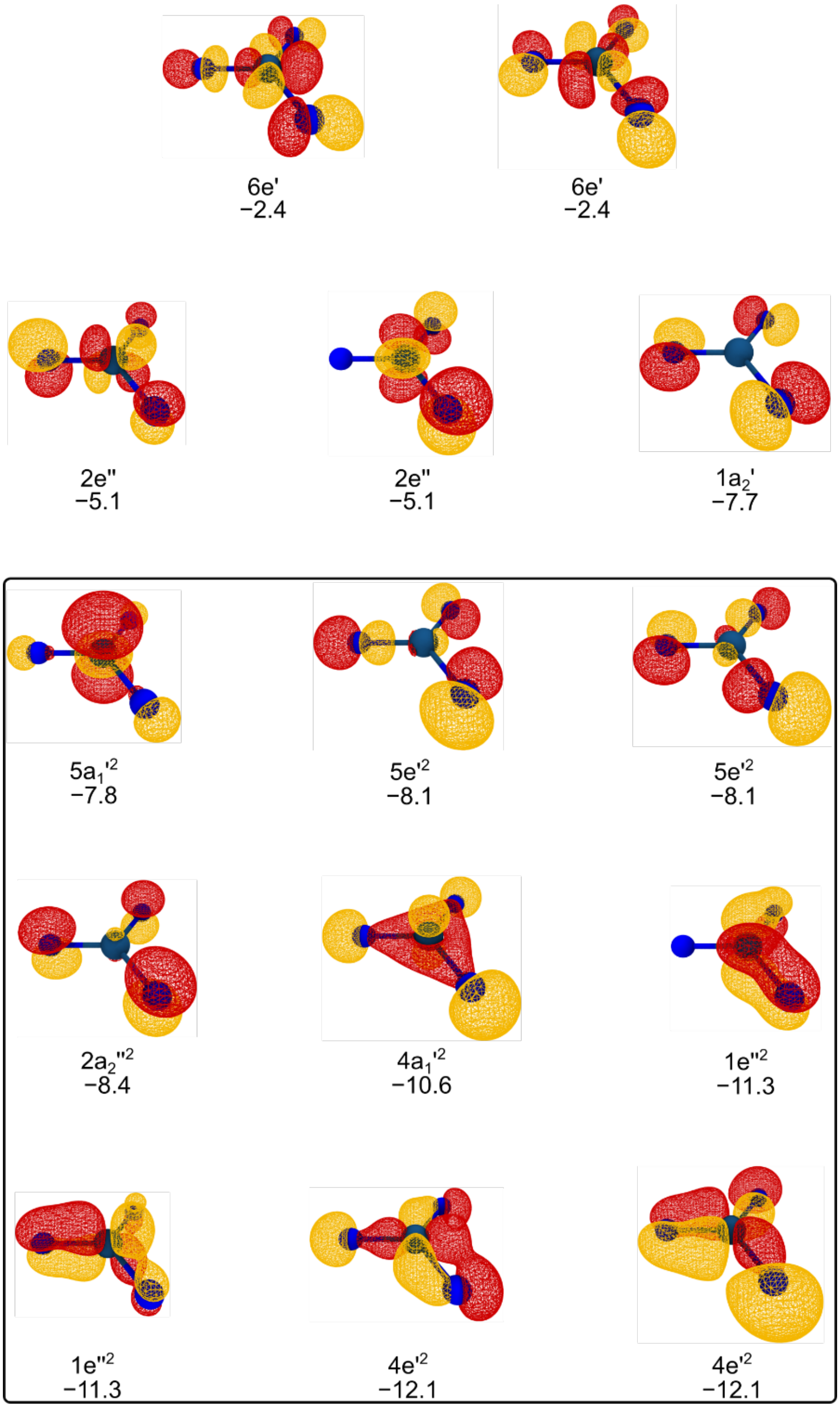

Figure 6. Molecular orbitals arising from the 5d(Ir) and 2p(N) atomic orbitals of $Ir(N)_3$ calculated at the R-BP86/ZORA-def2-TZVPP(N)/SARC-ZORA-TZVPP(Ir) level of theory with corresponding symmetries and energies (eV). Occupation numbers of orbitals inside the box 2, outside 0.

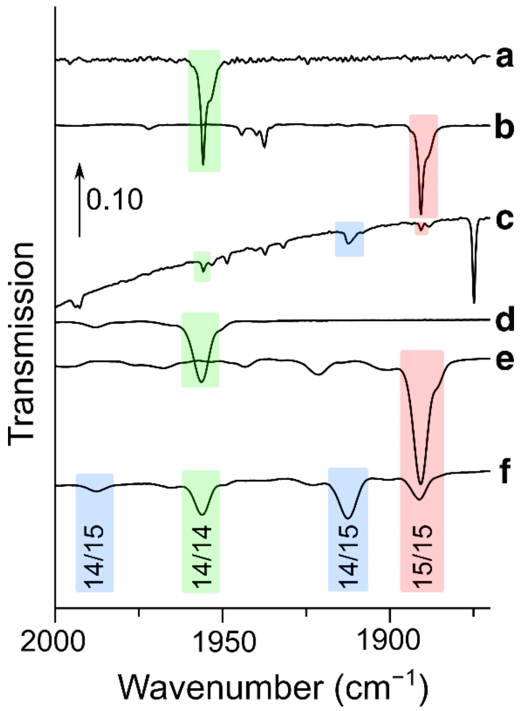

Figure S7. Infrared transmission spectrum of $Ir(N_2)^-$ in the 1870–2000 cm^{-1} region obtained by reacting laser-ablated iridium atoms with neat $^{14}N_2$ (**a**), neat $^{15}N_2$ (**b**), a neat 1:1 mixture of $^{14}N_2$ and $^{15}N_2$ (**c**), 10 % of $^{14}N_2$ in Ne (**d**), 10 % of $^{15}N_2$ in Ne (**e**) and 10 % of a 1:1 mixture of $^{14}N_2$ and $^{15}N_2$ in Ne (**f**). Trace **c** is enhanced by a factor of 6. The highlighted bands are assigned to the isotopologues $Ir(^{14}N)_2(^{14}N_2)$ (green), $Ir(^{14}N)_2(^{15}N_2)$ (blue), and $Ir(^{15}N)_2(^{15}N_2)$ (red).

Supplemental computational results

The vibrational data were calculated with the mass of the most abundant isotope, unless indicated otherwise. Vibrational modes with wavenumbers equal to zero (rotations and translations) are omitted. Point groups and electronic states given here are the ones used in the calculations. The electronic energy is given in hartree, x y z coordinates in Angstroem. Unless otherwise indicated, all BP86 and M06-L DFT calculations were carried out using the unrestricted Kohn-Sham (UKS) formalism.

BP86/def2-QZVP

N_2 (D_{6h}, $^1A_{1g}$) RKS

```
Energy = -109.5871592871
N     0.0000000    0.0000000    0.5508504
N     0.0000000    0.0000000   -0.5508504
```

$^{14}N_2$

```
#  mode     symmetry     wave number   IR intensity    selection rules
#                         cm**(-1)        km/mol         IR     RAMAN
     6        a1g          2347.76         0.00000       NO      YES
```

$^{14}N^{15}N$ ($^1A_1$)

```
#                         cm**(-1)        km/mol         IR     RAMAN
     6        a1           2308.70         0.00000       YES     YES
```

$^{15}N_2$

```
#  mode     symmetry     wave number   IR intensity    selection rules
#                         cm**(-1)        km/mol         IR     RAMAN
     6        a1g          2268.70         0.00000       NO      YES
```

N_3 (D_{6h}, $^2B_{3g}$)

```
Energy = -164.2411958613
N     0.0000000    0.0000000    1.1852937
N     0.0000000    0.0000000    0.0000000
N     0.0000000    0.0000000   -1.1852937
```

$^{14}N_3$

```
#  mode     symmetry     wave number   IR intensity    selection rules
#                         cm**(-1)        km/mol         IR     RAMAN
     6        b2u           478.69         9.06192       YES     NO
     7        b3u           583.88         1.25736       YES     NO
     8        ag           1318.96         0.00000       NO      YES
     9        b1u          1716.01       141.84904       YES     NO
```

N_3^- (D_{6h}, $^1A_{1g}$) RKS

```
Energy = -164.3423630893
N     0.0000000    0.0000000    1.1914452
N     0.0000000    0.0000000   -0.0000000
N     0.0000000    0.0000000   -1.1914452
```

$^{14}N_3^-$

```
#  mode     symmetry     wave number   IR intensity    selection rules
#                         cm**(-1)        km/mol         IR     RAMAN
     6        e1u           630.52         1.45779       YES     NO
     7        e1u           630.52         1.45779       YES     NO
     8        a1g          1296.02         0.00000       NO      YES
     9        a2u          2042.07       893.58204       YES     NO
```

$^{15}N_3^-$

```
#  mode     symmetry     wave number   IR intensity    selection rules
#                         cm**(-1)        km/mol         IR     RAMAN
     6        e1u           609.28         1.36125       YES     NO
     7        e1u           609.28         1.36125       YES     NO
     8        a1g          1252.37         0.00000       NO      YES
     9        a2u          1973.30       834.40904       YES     NO
```

$^{14}N^{15}N^{15}N^-$ (C_{6v}, $^1A_1$)

```
#  mode     symmetry     wave number   IR intensity    selection rules
#                         cm**(-1)        km/mol         IR     RAMAN
     6        e1            612.87         1.56487       YES     YES
     7        e1            612.87         1.56487       YES     YES
     8        a1           1273.95         0.07689       YES     YES
     9        a1           1985.20       848.71210       YES     YES
```

$^{15}N^{14}N^{14}N^-$ (C_{6v}, $^1A_1$)

# mode	symmetry	wave number cm**(-1)	IR intensity km/mol	selection rules IR	RAMAN
6	e1	627.03	1.26587	YES	YES
7	e1	627.03	1.26587	YES	YES
8	a1	1273.98	0.05871	YES	YES
9	a1	2031.02	879.19016	YES	YES

IrN (C_{6v}, $^1A_1$)

```
Energy = -159.2161562580
Ir    0.0000000    0.0000000   -0.7990476
N     0.0000000    0.0000000    0.7990476
```

$Ir^{14}N$

# mode	symmetry	wave number cm**(-1)	IR intensity km/mol	selection rules IR	RAMAN
6	a1	1208.95	36.14598	YES	YES

$Ir^{15}N$

# mode	symmetry	wave number cm**(-1)	IR intensity km/mol	selection rules IR	RAMAN
6	a1	1171.04	33.91496	YES	YES

$Ir(N)_2$ (C_{2v}, $^2B_1$)

```
Energy = -213.9559837886
N     1.4123939    0.0000000    0.3160590
Ir    0.0000000    0.0000000   -0.6321178
N    -1.4123939    0.0000000    0.3160590
```

$Ir(^{14}N)_2$

# mode	symmetry	wave number cm**(-1)	IR intensity km/mol	selection rules IR	RAMAN
7	a1	392.69	0.02446	YES	YES
8	b1	868.62	41.44662	YES	YES
9	a1	1027.06	1.51089	YES	YES

$Ir(^{15}N)_2$

# mode	symmetry	wave number cm**(-1)	IR intensity km/mol	selection rules IR	RAMAN
7	a1	380.51	0.02324	YES	YES
8	b1	842.08	39.04173	YES	YES
9	a1	994.21	1.41392	YES	YES

$Ir(^{15}N)(^{14}N)$ (C_s, A’’)

# mode	symmetry	wave number cm**(-1)	IR intensity km/mol	selection rules IR	RAMAN
7	a'	387.40	0.03082	YES	YES
8	a'	852.88	39.69499	YES	YES
9	a'	1011.80	1.91496	YES	YES

$Ir(N)_2$ (C_{2v}, $^2B_2$)

```
Energy = -213.9168489215
N     1.4829328    0.0000000    0.2901401
Ir    0.0000000    0.0000000   -0.5802799
N    -1.4829328    0.0000000    0.2901401
```

# mode	symmetry	wave number cm**(-1)	IR intensity km/mol	selection rules IR	RAMAN
7	a1	276.66	0.58989	YES	YES
8	a1	983.51	6.92432	YES	YES
9	b1	1119.44	595.71207	YES	YES

$Ir(N)_2$ (C_{2v}, $^2A_1$)

```
Energy = -213.9140155669
N     1.4875439    0.0000000    0.2814508
Ir    0.0000000    0.0000000   -0.5629013
N    -1.4875439    0.0000000    0.2814508
```

# mode	symmetry	wave number cm**(-1)	IR intensity km/mol	selection rules IR	RAMAN
7	a1	346.49	0.80753	YES	YES
8	a1	990.22	1.15859	YES	YES
9	b1	1004.94	3.37700	YES	YES

Ir(N)$_2$ (C_{2v}, $^4A_2$)

```
Energy = -213.9321859773
N     1.5245185    0.0000000    0.2729119
Ir    0.0000000    0.0000000   -0.5458234
N    -1.5245185    0.0000000    0.2729119
```

# mode	symmetry	wave number	IR intensity	selection rules	
#		cm**(-1)	km/mol	IR	RAMAN
7	a1	254.36	1.92459	YES	YES
8	b1	466.21	33.49477	YES	YES
9	a1	957.58	11.11216	YES	YES

Ir(N)$_3$ (D_{3h}, $^1A_1$')

```
Energy = -268.6541880303
Ir    0.0000000    0.0000000    0.0000000
N     0.8805308   -1.5251241    0.0000000
N     0.8805308    1.5251241    0.0000000
N    -1.7610616    0.0000000    0.0000000
```

# mode	symmetry	wave number	IR intensity	selection rules	
#		cm**(-1)	km/mol	IR	RAMAN
7	a2"	134.71	0.98897	YES	NO
8	e'	185.11	0.61870	YES	YES
9	e'	185.11	0.61870	YES	YES
10	e'	770.49	0.69120	YES	YES
11	e'	770.49	0.69120	YES	YES
12	a1'	968.10	0.00000	NO	YES

Ir(N$_2$) (C_{6v}, $^2A_2$)

```
Energy = -214.0303648898
Ir    0.0000000    0.0000000   -1.5731627
N     0.0000000    0.0000000    0.2208581
N     0.0000000    0.0000000    1.3523046
```

Ir($^{14}N_2$)

# mode	symmetry	wave number	IR intensity	selection rules	
#		cm**(-1)	km/mol	IR	RAMAN
6	b1	381.97	2.82388	YES	YES
7	b2	381.97	2.82388	YES	YES
8	a1	584.12	9.63272	YES	YES
9	a1	2119.09	282.87333	YES	YES

Ir($^{15}N_2$)

# mode	symmetry	wave number	IR intensity	selection rules	
#		cm**(-1)	km/mol	IR	RAMAN
6	b2	368.85	2.63819	YES	YES
7	b2	368.85	2.63819	YES	YES
8	a1	566.85	9.07778	YES	YES
9	a1	2047.34	264.17935	YES	YES

Ir(N$_2$) (C_s, ^4A')

```
Energy = -213.9887166667
N    -0.1306671   -0.3041779    0.0000000
N     0.1114863   -1.4013518    0.0000000
Ir    0.0191808    1.7055297    0.0000000
```

# mode	symmetry	wave number	IR intensity	selection rules	
#		cm**(-1)	km/mol	IR	RAMAN
7	a'	142.43	19.57173	YES	YES
8	a'	332.09	0.12137	YES	YES
9	a'	2118.96	311.72410	YES	YES

Ir(N$_2$)$^-$ (C_{2v}, $^1A_1$)

```
Energy = -214.0996310483
```

Ir	0.0000000	0.0000000	-1.5443968
N	0.0000000	0.0000000	0.1926815
N	0.0000000	0.0000000	1.3517153

Ir($^{14}N_2$)$^-$

# mode	symmetry	wave number	IR intensity	selection rules	
#		cm**(-1)	km/mol	IR	RAMAN
6	b1	429.13	2.65017	YES	YES
7	b2	429.13	2.65017	YES	YES
8	a1	653.87	5.31846	YES	YES
9	a1	1954.76	675.12711	YES	YES

Ir($^{15}N_2$)$^-$

# mode	symmetry	wave number	IR intensity	selection rules	
#		cm**(-1)	km/mol	IR	RAMAN
6	b2	414.80	2.43853	YES	YES
7	b2	414.80	2.43853	YES	YES
8	a1	634.68	5.21638	YES	YES
9	a1	1888.98	630.72998	YES	YES

Ir(N_2)$^+$ (C_s, $^4A'$)

Energy = -213.7070345445

Ir	0.0000000	0.0000000	-1.6178553
N	0.0000000	0.0000000	0.2509589
N	0.0000000	0.0000000	1.3668964

Ir($^{14}N_2$)$^+$

# mode	symmetry	wave number	IR intensity	selection rules	
#		cm**(-1)	km/mol	IR	RAMAN
6	b2	341.18	0.90551	YES	YES
7	b2	341.18	0.90551	YES	YES
8	a1	487.91	15.23200	YES	YES
9	a1	2211.96	48.49148	YES	YES

Ir($^{15}N_2$)$^+$

# mode	symmetry	wave number	IR intensity	selection rules	
#		cm**(-1)	km/mol	IR	RAMAN
6	b2	329.77	0.86805	YES	YES
7	b2	329.77	0.86805	YES	YES
8	a1	473.60	13.99107	YES	YES
9	a1	2137.47	45.27682	YES	YES

Ir(N_2)$_2$ (D_{2h}, ^2A_g)

Energy = -323.6864664802

Ir	0.0000000	0.0000000	0.0000000
N	0.0000000	0.0000000	1.8991645
N	0.0000000	0.0000000	3.0198683
N	0.0000000	0.0000000	-1.8991645
N	0.0000000	0.0000000	-3.0198683

Ir($^{14}N_2$)$_2$

# mode	symmetry	wave number	IR intensity	selection rules	
#		cm**(-1)	km/mol	IR	RAMAN
6	b3u	75.49	0.27235	YES	NO
7	b2u	75.50	0.27235	YES	NO
8	b3g	326.53	0.00000	NO	YES
9	b2g	326.54	0.00000	NO	YES
10	b1u	439.41	99.53577	YES	NO
11	b3u	463.30	0.03105	YES	NO
12	b2u	463.35	0.03085	YES	NO
13	ag	490.52	0.00000	NO	YES
14	b1u	2148.81	1001.24580	YES	NO
15	ag	2200.37	0.00000	NO	YES

Ir($^{15}N_2$)$_2$

# mode	symmetry	wave number	IR intensity	selection rules	
#		cm**(-1)	km/mol	IR	RAMAN
6	b3u	73.42	0.25760	YES	NO
7	b2u	73.43	0.25760	YES	NO
8	b3g	315.54	0.00000	NO	YES
9	b2g	315.55	0.00000	NO	YES
10	b1u	428.00	94.43042	YES	NO
11	b3u	448.36	0.03018	YES	NO
12	b2u	448.40	0.03000	YES	NO
13	ag	474.00	0.00000	NO	YES
14	b1u	2076.44	934.98223	YES	NO
15	ag	2126.27	0.00000	NO	YES

Ir($^{15}N_2$)($^{14}N_2$) (C_{2v}, $^2A_1$)

# mode	symmetry	wave number	IR intensity	selection rules	
#		cm**(-1)	km/mol	IR	RAMAN
6	b1	74.46	0.26496	YES	YES

7	b2	74.47	0.26495	YES	YES
8	b2	320.74	0.00010	YES	YES
9	b1	320.75	0.00010	YES	YES
10	a1	432.74	94.71981	YES	YES
11	b1	456.14	0.03053	YES	YES
12	b2	456.18	0.03034	YES	YES
13	a1	483.23	2.26519	YES	YES
14	a1	2093.45	745.54068	YES	YES
15	a1	2182.49	222.57143	YES	YES

$Ir(N_2)_2$ (D_{2h}, $^4B_{2u}$)

```
Energy = -323.5917128454
Ir    0.0000000    0.0000000    0.0000000
N     0.0000000    0.0000000    1.9197455
N     0.0000000    0.0000000    3.0595303
N     0.0000000    0.0000000   -1.9197455
N     0.0000000    0.0000000   -3.0595303
```

# mode	symmetry	wave number	IR intensity	selection rules	
#		cm**(-1)	km/mol	IR	RAMAN
6	b2u	87.71	2.25204	YES	NO
7	b3u	95.54	1.80407	YES	NO
8	b3u	276.08	29.91069	YES	NO
9	b3g	315.71	0.00000	NO	YES
10	b2g	368.99	0.00000	NO	YES
11	b1u	443.80	0.04404	YES	NO
12	ag	475.61	0.00000	NO	YES
13	b2u	493.01	0.12332	YES	NO
14	b1u	1963.81	1793.21471	YES	NO
15	ag	2055.51	0.00000	NO	YES

$Ir(N_2)_2^+$ (D_{2h}, ^3A_g)

```
Energy = -323.3643926816
Ir    0.0000000    0.0000000    0.0000000
N     0.0000000    0.0000000    1.9555958
N     0.0000000    0.0000000    3.0659497
N     0.0000000    0.0000000   -1.9555958
N     0.0000000    0.0000000   -3.0659497
```

# mode	symmetry	wave number	IR intensity	selection rules	
#		cm**(-1)	km/mol	IR	RAMAN
6	b2u	85.52	0.10171	YES	NO
7	b3u	85.52	0.10171	YES	NO
8	b2g	304.51	0.00000	NO	YES
9	b3g	304.51	0.00000	NO	YES
10	b1u	383.06	48.38707	YES	NO
11	ag	430.73	0.00000	NO	YES
12	b2u	470.20	0.61928	YES	NO
13	b3u	470.20	0.61928	YES	NO
14	b1u	2242.64	173.28622	YES	NO
15	ag	2269.64	0.00000	NO	YES

$Ir(N_2)_2^+$ (D_{2h}, ^1A_g)

```
Energy = -323.3568461762
Ir    0.0000000    0.0000000    0.0000000
N     0.0000000    0.0000000    1.9524098
N     0.0000000    0.0000000    3.0630648
N     0.0000000    0.0000000   -1.9524098
N     0.0000000    0.0000000   -3.0630648
```

# mode	symmetry	wave number	IR intensity	selection rules	
#		cm**(-1)	km/mol	IR	RAMAN
6	b2u	85.75	0.09727	YES	NO
7	b3u	85.75	0.09727	YES	NO
8	b2g	307.75	0.00000	NO	YES
9	b3g	307.75	0.00000	NO	YES
10	b1u	382.67	47.66188	YES	NO
11	ag	434.27	0.00000	NO	YES
12	b3u	477.52	0.71588	YES	NO
13	b2u	477.52	0.71589	YES	NO
14	b1u	2239.14	173.80930	YES	NO
15	ag	2265.58	0.00000	NO	YES

$Ir(N_2)_2^+$ (D_{2h}, ^1A_g)

```
Energy = -323.3272925494
Ir    0.0000000    0.0000000    0.0000000
N     0.0000000    0.0000000    1.9378028
N     0.0000000    0.0000000    3.0506974
N     0.0000000    0.0000000   -1.9378028
```

```
N     0.0000000    0.0000000   -3.0506974
```

# mode	symmetry	wave number cm**(-1)	IR intensity km/mol	selection rules IR	RAMAN
6	e1u	84.33	0.02829	YES	NO
7	e1u	84.33	0.02829	YES	NO
8	e1g	318.71	0.00000	NO	YES
9	e1g	318.71	0.00000	NO	YES
10	a2u	378.50	46.34882	YES	NO
11	a1g	451.74	0.00000	NO	YES
12	e1u	507.55	0.81635	YES	NO
13	e1u	507.55	0.81635	YES	NO
14	a2u	2209.40	211.08150	YES	NO
15	a1g	2244.15	0.00000	NO	YES

$Ir(N_2)_2^-$ (C_{2v}, $^1A_1$)

```
Energy = -323.7712152965
N    -1.8251145    0.0000000    0.0646127
N    -2.9215603    0.0000000   -0.2597363
Ir    0.0000000    0.0000000    0.3902468
N     1.8251145    0.0000000    0.0646127
N     2.9215603    0.0000000   -0.2597363
```

$Ir(^{14}N_2)_2^-$

# mode	symmetry	wave number cm**(-1)	IR intensity km/mol	selection rules IR	RAMAN
7	a1	68.16	3.24006	YES	YES
8	a2	353.25	0.00000	NO	YES
9	b1	362.69	0.05866	YES	YES
10	b2	441.59	0.96035	YES	YES
11	b1	486.15	68.36593	YES	YES
12	a1	493.22	3.16098	YES	YES
13	a1	547.61	0.55652	YES	YES
14	b1	1988.14	1926.19429	YES	YES
15	a1	2051.53	37.69070	YES	YES

$Ir(^{15}N_2)_2^-$

# mode	symmetry	wave number cm**(-1)	IR intensity km/mol	selection rules IR	RAMAN
7	a1	66.27	2.89115	YES	YES
8	a2	341.36	0.00000	NO	YES
9	b1	350.48	0.05370	YES	YES
10	b2	427.15	0.88392	YES	YES
11	b1	473.41	65.70096	YES	YES
12	a1	477.44	2.91609	YES	YES
13	a1	529.19	0.51669	YES	YES
14	b1	1921.19	1798.87534	YES	YES
15	a1	1982.44	35.20007	YES	YES

$Ir(^{15}N_2)(^{14}N_2)^-$ (Cs, $^1A'$)

# mode	symmetry	wave number cm**(-1)	IR intensity km/mol	selection rules IR	RAMAN
7	a	67.22	3.06527	YES	YES
8	a	346.81	0.00387	YES	YES
9	a	356.22	0.06184	YES	YES
10	a	434.86	0.91828	YES	YES
11	a	478.08	59.35779	YES	YES
12	a	486.49	9.59787	YES	YES
13	a	539.32	1.65117	YES	YES
14	a	1939.72	1541.85010	YES	YES
15	a	2031.93	357.12640	YES	YES

$Ir(N_3)$ (C_s, $^3A''$)

```
Energy = -268.7297430506
Ir   -0.8727706    1.8857263    0.0000000
N    -0.7277581    0.0225820    0.0000000
N     0.3642280   -0.5803123    0.0000000
N     1.2363008   -1.3279960    0.0000000
```

# mode	symmetry	wave number cm**(-1)	IR intensity km/mol	selection rules IR	RAMAN
7	a'	143.79	3.56380	YES	YES
8	a'	430.61	3.54809	YES	YES
9	a"	443.15	1.37139	YES	YES
10	a'	671.92	5.10472	YES	YES
11	a'	1152.81	53.23297	YES	YES
12	a'	2019.81	407.41122	YES	YES

IrNNIr (D_{2h}, $^3B_{1u}$)

```
Energy = -318.4719683227
```

```
Ir    0.0000000    0.0000000   -2.3766401
N     0.0000000    0.0000000   -0.5754674
N     0.0000000    0.0000000    0.5754674
Ir    0.0000000    0.0000000    2.3766401
```

$Ir^{14}N^{14}NIr$

# mode	symmetry	wave number cm**(-1)	IR intensity km/mol	selection rules IR	RAMAN
6	b2u	111.33	6.24364	YES	NO
7	b3u	111.33	6.24364	YES	NO
8	ag	203.20	0.00000	NO	YES
9	b3g	256.80	0.00000	NO	YES
10	b2g	256.80	0.00000	NO	YES
11	b1u	781.81	161.57713	YES	NO
12	ag	2080.71	0.00000	NO	YES

$Ir^{15}N^{15}NIr$

# mode	symmetry	wave number cm**(-1)	IR intensity km/mol	selection rules IR	RAMAN
6	b2u	107.84	5.85827	YES	NO
7	b3u	107.84	5.85827	YES	NO
8	ag	203.20	0.00000	NO	YES
9	b3g	248.19	0.00000	NO	YES
10	b2g	248.19	0.00000	NO	YES
11	b1u	757.29	151.60420	YES	NO
12	ag	2010.69	0.00000	NO	YES

$Ir^{14}N^{15}NIr$ (C_{2v}, $^3A_1$)

# mode	symmetry	wave number cm**(-1)	IR intensity km/mol	selection rules IR	RAMAN
6	b2	109.52	6.04099	YES	YES
7	b1	109.52	6.04099	YES	YES
8	a1	203.20	0.00000	YES	YES
9	b2	252.57	0.00997	YES	YES
10	b1	252.57	0.00997	YES	YES
11	a1	769.16	156.35928	YES	YES
12	a1	2046.18	0.23139	YES	YES

NIr(N_2) (C_s, $^1A'$)

```
Energy = -268.8194957881
Ir   -1.0046934   -0.4350909    0.0000000
N    -0.6944564   -2.0561243    0.0000000
N     0.3500444    0.9867359    0.0000000
N     1.3491054    1.5044794    0.0000000
```

$^{14}NIr(^{14}N_2)$

# mode	symmetry	wave number cm**(-1)	IR intensity km/mol	selection rules IR	RAMAN
7	a'	146.66	7.90813	YES	YES
8	a"	310.09	1.70767	YES	YES
9	a'	342.87	4.01490	YES	YES
10	a'	417.83	3.83047	YES	YES
11	a'	1084.86	27.23003	YES	YES
12	a'	2109.59	388.12093	YES	YES

$^{15}NIr(^{14}N_2)$

# mode	symmetry	wave number cm**(-1)	IR intensity km/mol	selection rules IR	RAMAN
7	a'	143.44	7.46759	YES	YES
8	a"	309.89	1.73449	YES	YES
9	a'	342.83	3.96068	YES	YES
10	a'	416.59	4.05497	YES	YES
11	a'	1050.80	25.53628	YES	YES
12	a'	2109.57	388.23032	YES	YES

IrIrN (C_s, $^2A'$)

```
Energy = -263.7144182651
Ir   -0.5963376    1.8879672    0.0000000
Ir   -0.3190028   -0.3927475    0.0000000
N     0.9153404   -1.4952197    0.0000000
```

$IrIr^{14}N$

# mode	symmetry	wave number cm**(-1)	IR intensity km/mol	selection rules IR	RAMAN
7	a'	105.80	5.85343	YES	YES
8	a'	221.76	0.95737	YES	YES
9	a'	1054.29	60.02501	YES	YES

$IrIr^{15}N$

# mode	symmetry	wave number cm**(-1)	IR intensity km/mol	selection rules IR	RAMAN
7	a'	102.86	5.46297	YES	YES

```
     8        a'            221.66         0.95186       YES     YES
     9        a'           1021.00        56.40977       YES     YES
```

BP86/ZORA-def2-TZVPP(N)/SARC-ZORA-TZVPP(Ir)

Ir$(N)_2$ (C_1, ^2A)

```
Energy = -18527.459076532468
Ir  -0.00000005284280      0.00000000000000     -0.63128496123684
N   -1.41022715939508      0.00000000000000      0.31564266296423
N    1.41022721223788      0.00000000000000      0.31564269827261

 Mode    freq (cm**-1)   T**2         TX         TY         TZ
-------------------------------------------------------------------
   6:       392.86    0.015350  ( -0.000000   0.000000  -0.123897)
   7:       878.80   37.913039  ( -6.157356  -0.000000   0.000007)
   8:      1035.26    1.480248  ( -0.000023   0.000000   1.216654)
```

Ir$(N)_2$ TS (C_1, ^2A)

```
Energy = -18527.365959366154
Ir  -0.03866148720625      0.00000000000000     -1.00023248877844
N   -0.97294630786287      0.00000000000000      0.47158165101008
N    1.01160779506913      0.00000000000000      0.52865183776837

 Mode    freq (cm**-1)   T**2         TX         TY         TZ
-------------------------------------------------------------------
   6:      -736.33    ***imaginary mode***
   7:       431.17    0.674613  ( -0.407012  -0.000001  -0.713410)
   8:       908.13    1.997347  (  0.615350  -0.000000  -1.272278)
```

Ir$(N)_2$ TS (C_1, ^4A)

```
Energy = -18527.361054029960
Ir   0.03301036678652      0.00000000000000     -1.00750169971361
N   -0.98054236764465      0.00000000000000      0.47425130571116
N    0.94753200085812      0.00000000000000      0.53325139400246

 Mode    freq (cm**-1)   T**2         TX         TY         TZ
-------------------------------------------------------------------
   6:     -2308.50    ***imaginary mode***
   7:       367.41    1.577166  (  1.255181   0.034555   0.022207)
   8:       893.08    2.833148  ( -0.033693  -0.348179   1.646446)
```

Ir(N_2) (C_1, ^2A)

```
Energy = -18527.508956688940
Ir  -0.00000000066953     -0.00000000031987     -1.56484112101323
N   0.00000000133903      0.00000000063987      0.21341197152127
N   -0.00000000066950     -0.00000000031999      1.35142914949195

 Mode    freq (cm**-1)   T**2         TX         TY         TZ
-------------------------------------------------------------------
   5:       392.74    1.514507  (  0.548736   1.101542  -0.000000)
   6:       392.74    1.514508  (  1.101542  -0.548737   0.000000)
   7:       602.92   11.393393  (  0.000000   0.000000  -3.375410)
   8:      2074.84  327.932089  ( -0.000000  -0.000000  18.108895)
```

Ir$(N)_3$ (C_1, ^1A) RKS

```
Energy = -18582.208568531445
N       0.877993      1.520731     -0.000000
Ir     -0.000001      0.000000      0.000000
N       0.877993     -1.520731     -0.000000
N      -1.755985      0.000000     -0.000000

 Mode    freq (cm**-1)   T**2         TX         TY         TZ
-------------------------------------------------------------------
   6:       142.84    0.795457  (  0.000000   0.000000   0.891884)
   7:       189.97    0.508095  ( -0.712808   0.000045  -0.000000)
   8:       190.14    0.507954  (  0.000045   0.712709  -0.000000)
   9:       788.71    0.688991  (  0.830055   0.000374  -0.000000)
  10:       788.72    0.688937  ( -0.000374   0.830022  -0.000000)
  11:       976.94    0.000000  (  0.000059   0.000000   0.000000)
```

Ir$(N)_3$ (C_1, ^1A)

```
Energy = -18582.208568533388
N       0.877996      1.520736      0.000000
Ir     -0.000002      0.000000     -0.000000
```

```
N       0.877996     -1.520736      0.000000
N      -1.755990      0.000000      0.000000

 Mode    freq (cm**-1)   T**2         TX         TY         TZ
-------------------------------------------------------------------
   6:       142.86    0.795777  ( -0.000000   0.000000  -0.892063)
   7:       189.98    0.508199  (  0.712881   0.000024   0.000000)
   8:       190.15    0.507974  ( -0.000029   0.712723  -0.000000)
   9:       788.70    0.689450  (  0.830266  -0.010414  -0.000000)
  10:       788.70    0.689159  (  0.010430   0.830091  -0.000000)
  11:       976.93    0.000000  (  0.000075  -0.000010   0.000000)
```

Ir(N)$_3$ TS (C$_1$, ^{1}A) RKS

```
Energy = -18582.191698412571
N    0.98504117841954      1.70618191713348     -0.01726953128539
Ir   0.13626371759078      0.23596137442520     -0.00256235402343
N    0.41847717313611     -1.53641771301031      0.00986257570181
N   -1.53978106914643     -0.40572557854836      0.00996930960701

 Mode    freq (cm**-1)   T**2         TX         TY         TZ
-------------------------------------------------------------------
   6:      -315.28    ***imaginary mode***
   7:       123.26    1.381596  ( -0.001127  -0.010960  -1.175362)
   8:       189.88    5.598902  ( -2.049855   1.181945  -0.002140)
   9:       799.34    0.086670  ( -0.255809   0.145710   0.000010)
  10:       857.65   28.574006  (  2.672182   4.629386  -0.047255)
  11:      1000.79   14.419617  ( -1.898642  -3.288417   0.032987)
```

Ir(N)$_3$ TS (C$_1$, ^{3}A)

```
Energy = -18582.165316776885
N   0.89916651760164      1.62753405912247      0.20507888528382
Ir  0.23407666406960      0.21539804792815     -0.48693024802453
N   0.32738784340781     -1.41585703748299      0.16654561326475
N   -1.46063002507905     -0.42707506956763      0.11530574947595

 Mode    freq (cm**-1)   T**2         TX         TY         TZ
-------------------------------------------------------------------
   6:      -689.42    ***imaginary mode***
   7:       213.83    3.072218  (  1.727321  -0.191056   0.228209)
   8:       246.22    9.319231  (  0.115990  -0.589404   2.993055)
   9:       587.63    4.374417  (  1.554629  -1.337964   0.409143)
  10:       865.97   20.637535  ( -0.849950  -4.308688  -1.162034)
  11:       972.20    8.060881  (  1.123561   2.607322   0.019021)
```

Ir(N)(N$_2$) (C$_1$, ^{3}A) RKS

```
Energy =
Ir  -0.99843601031668     -0.43465654378306      0.00001460729074
N   -0.69857605006467     -2.05566281879788     -0.00000414845203
N   0.35043497938319      0.98244325687028     -0.00002586909360
N   1.34657708099816      1.50787610571065      0.00001541025489

 Mode    freq (cm**-1)   T**2         TX         TY         TZ
------------------------------------------------------------------
   6:       149.71    7.640629  (  2.542583   1.084390  -0.000051)
   7:       315.22    1.726735  (  0.000292   0.000128  -1.314053)
   8:       349.88    3.848438  (  1.712748   0.956521   0.000004)
   9:       422.49    4.003985  ( -1.792344  -0.889656   0.000145)
  10:      1095.70   25.572155  (  4.370009  -2.544636  -0.000227)
  11:      2109.71  378.724859  ( 14.037302  13.478836  -0.000371)
```

M06-L/ZORA-def2-TZVPP(N)/SARC-ZORA-TZVPP(Ir)

Ir(N)$_2$ (C$_1$, ^{2}A)

```
Energy = -18525.467056933678
Ir  -0.00000009045772      0.00000000000000     -0.62823498277550
N   -1.40069500576939      0.00000000000000      0.31411766236377
N    1.40069509622710      0.00000000000000      0.31411772041173

 Mode    freq (cm**-1)   T**2         TX         TY         TZ
-------------------------------------------------------------------
   6:       406.40    0.119210  (  0.005129  -0.000348  -0.345230)
   7:       875.66   48.032941  ( -6.930580  -0.000055   0.000232)
   8:      1058.48    3.434843  (  0.002007  -0.000050   1.853332)
```

Ir(N)$_2$ TS (C$_1$, ^{2}A)

```
Energy = -18525.372866912498
```

```
Ir  -0.05423592809509      0.00000000000000     -1.00715026943836
N   -0.95916993255531      0.00000000000000      0.46383141403110
N    1.01340586065041      0.00000000000000      0.54331985540728

 Mode    freq (cm**-1)   T**2         TX         TY         TZ
-------------------------------------------------------------------
   6:      -711.55    ***imaginary mode***
   7:       437.25    0.311448  (  0.545333   0.000038   0.118574)
   8:       911.08    4.595018  (  0.881119   0.000033  -1.954136)
```

Ir(N)$_3$ (C$_1$, ^{1}A) RKS

```
Energy = -18580.193789971730
N    0.87443135203633      1.51461844965352      0.00000094120495
Ir  -0.00003416411595     -0.00000030039386     -0.00000282342226
N    0.87443065827745     -1.51461784888252      0.00000094120706
N   -1.74882784619783     -0.00000030037713      0.00000094101025

 Mode    freq (cm**-1)   T**2         TX         TY         TZ
-------------------------------------------------------------------
   6:       126.46    1.535762  ( -0.010504   0.007064  -1.239194)
   7:       193.03    1.102940  ( -0.799239   0.681121   0.015210)
   8:       195.47    1.122133  ( -0.655602  -0.832056   0.001262)
   9:       773.87    0.054071  ( -0.232480   0.004928  -0.000142)
  10:       776.07    0.086261  ( -0.007649  -0.293597   0.001649)
  11:      1003.90    0.000467  ( -0.020525   0.006719   0.000341)
```

Ir(N)$_3$ TS (C$_1$, ^{1}A) RKS

```
Energy = -18580.178264754060
N   0.97490410868930      1.68881126151210     -0.01770995567920
Ir  0.13030719215327      0.22545450004475      0.00043654942375
N   0.44375596989281     -1.53243636509665      0.00856198738820
N   -1.54896627073538     -0.38182939646019      0.00871141886726

 Mode    freq (cm**-1)   T**2         TX         TY         TZ
-------------------------------------------------------------------
   6:      -306.08    ***imaginary mode***
   7:       105.94    2.400416  ( -0.082091  -0.019473  -1.547029)
   8:       184.62    8.962498  ( -2.605275   1.474798  -0.003351)
   9:       819.45    0.035455  ( -0.030187   0.185858  -0.000671)
  10:       869.51   38.409588  (  3.103273   5.364424  -0.047283)
  11:      1023.99   13.526293  ( -1.835324  -3.186866   0.041968)
```

CCSD(T)/aug-cc-pVTZ(-PP)

IrN (C$_{2v}$, ^{1}A$_1$)

```
Energy = -158.596318228943
Ir         0.00000        0.00000        0.10821
N          0.00000        0.00000       -1.49116


                     Normal Coordinate Analysis
  ----------------------------------------------------------------
    Irreducible         Harmonic       Infrared        Type
   Representation       Frequency      Intensity
  ----------------------------------------------------------------
                       (cm-1)         (km/mol)
  ----------------------------------------------------------------
       SG+             1195.3928        37.7081       VIBRATION
  ----------------------------------------------------------------
```

Ir(N$_2$) (C$_{2v}$, ^{2}A$_2$)

```
Energy = -213.296141642281
Ir     0.000000     0.000000     0.300969
 N     0.000000     0.000000    -1.511567
 N     0.000000     0.000000    -2.635797
```

Ir(^{14}N$_2$)

```
                     Normal Coordinate Analysis
  ----------------------------------------------------------------
    Irreducible         Harmonic       Infrared        Type
   Representation       Frequency      Intensity
  ----------------------------------------------------------------
                       (cm-1)         (km/mol)
  ----------------------------------------------------------------
        PI              377.1830         2.7636       VIBRATION
        PI              377.1830         2.7636       VIBRATION
       SG+              548.5365        29.0796       VIBRATION
       SG+             2140.2401       462.6555       VIBRATION
```

Ir($^{15}N_2$)

Normal Coordinate Analysis

Irreducible Representation	Harmonic Frequency (cm-1)	Infrared Intensity (km/mol)	Type
PI	364.5371	2.5891	VIBRATION
PI	364.5371	2.5891	VIBRATION
SG+	532.5155	27.4014	VIBRATION
SG+	2067.8968	431.9740	VIBRATION

Ir(N_2)$^-$ (C_{2v}, $^1A_1$)

```
Energy = -213.357526833504
Ir     0.000000     0.000000    -0.294152
 N     0.000000     0.000000     1.449982
 N     0.000000     0.000000     2.603441
```

Ir($^{14}N_2$)$^-$

Normal Coordinate Analysis

Irreducible Representation	Harmonic Frequency (cm-1)	Infrared Intensity (km/mol)	Type
PI	434.0213	3.3179	VIBRATION
PI	434.0213	3.3179	VIBRATION
SG+	648.4311	11.7478	VIBRATION
SG+	1962.7107	1017.9657	VIBRATION

Ir($^{15}N_2$)$^-$

Normal Coordinate Analysis

Irreducible Representation	Harmonic Frequency (cm-1)	Infrared Intensity (km/mol)	Type
PI	419.4812	3.1143	VIBRATION
PI	419.4812	3.1143	VIBRATION
SG+	629.4805	11.0542	VIBRATION
SG+	1896.4047	950.5014	VIBRATION

Ir(N_2)$^+$ (C_{2v}, $^3A_1$)

```
Energy = -212.992689411640
Ir     0.000000     0.000000    -0.315136
 N     0.000000     0.000000     1.617291
 N     0.000000     0.000000     2.725297
```

Ir($^{14}N_2$)$^+$

Normal Coordinate Analysis

Irreducible Representation	Harmonic Frequency (cm-1)	Infrared Intensity (km/mol)	Type
PI	313.1245	0.6018	VIBRATION
PI	313.1245	0.6018	VIBRATION
SG+	400.6114	14.3507	VIBRATION
SG+	2285.8884	23.5659	VIBRATION

Ir($^{15}N_2$)$^+$

Normal Coordinate Analysis

Irreducible Representation	Harmonic Frequency (cm-1)	Infrared Intensity (km/mol)	Type
PI	302.6168	0.5625	VIBRATION
PI	302.6168	0.5625	VIBRATION
SG+	388.9122	13.5247	VIBRATION
SG+	2208.6143	22.0039	VIBRATION

IrNNIr (D_{2h}, $^3B_{1u}$)

```
Energy = -317.213761825221
 N     0.000000     0.000000     0.569367
 N     0.000000     0.000000    -0.569367
Ir     0.000000     0.000000    -2.385426
Ir     0.000000     0.000000     2.385426
```

$Ir^{14}N^{14}NIr$

Normal Coordinate Analysis

Irreducible Representation	Harmonic Frequency (cm-1)	Infrared Intensity (km/mol)	Type
PIu	115.1277	5.7702	VIBRATION
PIu	115.1277	5.7702	VIBRATION
SGg+	194.6052	0.0000	VIBRATION
PIg	345.5060	0.0000	VIBRATION
PIg	345.5060	0.0000	VIBRATION
SGu-	729.1069	361.6940	VIBRATION
SGg+	2102.6390	0.0000	VIBRATION

$Ir^{15}N^{15}NIr$

Normal Coordinate Analysis

Irreducible Representation	Harmonic Frequency (cm-1)	Infrared Intensity (km/mol)	Type
PIu	111.5034	5.4126	VIBRATION
PIu	111.5034	5.4126	VIBRATION
SGg+	194.6019	0.0000	VIBRATION
PIg	333.8749	0.0000	VIBRATION
PIg	333.8749	0.0000	VIBRATION
SGu-	706.1538	339.2793	VIBRATION
SGg+	2031.5923	0.0000	VIBRATION

$Ir^{14}N^{15}NIr$ (C_{2v}, $^3A_1$)

Normal Coordinate Analysis

Irreducible Representation	Harmonic Frequency (cm-1)	Infrared Intensity (km/mol)	Type
PI	113.2602	5.5836	VIBRATION
PI	113.2602	5.5836	VIBRATION
SG+	194.6035	0.0000	VIBRATION
PI	339.7635	0.0077	VIBRATION
PI	339.7635	0.0077	VIBRATION
SG+	717.2738	349.9890	VIBRATION
SG+	2067.5765	0.4977	VIBRATION

References

[1] A. Citra, L. Andrews, *J. Phys. Chem. A* **2000**, *104*, 1152–1161.

[2] X. Wang, L. Andrews, *J. Phys. Chem. A* **2002**, *106*, 2457–2464.

[3] W. Klotzbuecher, G. A. Ozin, *J. Am. Chem. Soc.* **1975**, *97*, 2672–2675.

[4] A. Citra, X. Wang, W. D. Bare, L. Andrews, *J. Phys. Chem. A* **2001**, *105*, 7799–7811.
